# A high definition picture of somatic mutations in chronic lymphoproliferative disorder of natural killer cells

**DOI:** 10.1038/s41408-020-0309-2

**Published:** 2020-04-22

**Authors:** Vanessa Rebecca Gasparini, Andrea Binatti, Alessandro Coppe, Antonella Teramo, Cristina Vicenzetto, Giulia Calabretto, Gregorio Barilà, Annica Barizza, Edoardo Giussani, Monica Facco, Satu Mustjoki, Gianpietro Semenzato, Renato Zambello, Stefania Bortoluzzi

**Affiliations:** 1 Department of Medicine, Hematology and Clinical Immunology Branch, University of Padova, Padova, Italy; 2 Veneto Institute of Molecular Medicine (VIMM), Padova, Italy; 3 Department of Molecular Medicine, University of Padova, Padova, Italy; 4 Department of Women’s and Children’s Health, University of Padova, Padova, Italy; 5 Department of Biology, University of Padova, Padova, Italy; 6 Hematology Research Unit Helsinki, Helsinki University Hospital Comprehensive Cancer Center, Helsinki, Finland; 7 Translational Immunology Research Program and Department of Clinical Chemistry and Hematology, University of Helsinki, Helsinki, Finland; 8 CRIBI Interdepartmental Research Center for Innovative Biotechnologies (CRIBI), University of Padova, Padova, Italy

**Keywords:** Cancer genomics, Chronic lymphocytic leukaemia

## Abstract

The molecular pathogenesis of chronic lymphoproliferative disorder of natural killer (NK) cells (CLPD‐NK) is poorly understood. Following the screening of 57 CLPD-NK patients, only five presented *STAT3* mutations. WES profiling of 13 cases negative for *STAT3/STAT5B* mutations uncovered an average of 18 clonal, population rare and deleterious somatic variants per patient. The mutational landscape of CLPD-NK showed that most patients carry a heavy mutational burden, with major and subclonal deleterious mutations co-existing in the leukemic clone. Somatic mutations hit genes wired to cancer proliferation, survival, and migration pathways, in the first place Ras/MAPK, PI3K-AKT, in addition to JAK/STAT (*PIK3R1* and *PTK2*). We confirmed variants with putative driver role of *MAP10*, *MPZL1*, *RPS6KA1*, *SETD1B*, *TAOK2*, *TMEM127*, and *TNFRSF1A* genes, and of genes linked to viral infections (*DDX3X* and *RSF1*) and DNA repair (*PAXIP1*). A truncating mutation of the epigenetic regulator *TET2* and a variant likely abrogating *PIK3R1-*negative regulatory activity were validated. This study significantly furthered the view of the genes and pathways involved in CLPD-NK, indicated similarities with aggressive diseases of NK cells and detected mutated genes targetable by approved drugs, being a step forward to personalized precision medicine for CLPD-NK patients.

## Introduction

Among the abnormal proliferations of large granular lymphocytes (LGL), chronic lymphoproliferative disorder of natural killer (NK) cells (CLPD‐NK) is characterized by a persistent (>6 months) clonal expansion of LGL of the NK lineage^1^ in the peripheral blood with an absolute NK-LGL count ≥0.5 × 10^9^/L. CLPD‐NK patients are generally asymptomatic, with a minority of cases presenting with cytopenia, mainly neutropenia and anemia. CLPD‐NK is commonly associated with autoimmune disorders and neoplastic conditions, both hematological and non-hematological. The clinical course of CLPD-NK is usually indolent, consistent with the better characterized T-cell LGL leukemia (T-LGLL), whereas it is clearly distinguished from the aggressive NK cell leukemia (ANKL) and extranodal NK/T-cell lymphoma-nasal type (NKTCL). CLPD-NK is often characterized by a CD3− CD16+ CD56+ CD8± CD57± phenotype and a restricted pattern of killer immunoglobulin-like receptors (KIR) providing a surrogate marker of clonality^2^.

Evidence on the genomic landscape of T-LGLL pointed to a key role of somatic mutations within JAK/STAT (mostly in *STAT3* and *STAT5B* genes) and Ras/MAPK pathways^3–10^. In ANKL and NKTCL, *JAK2* and *STAT3* mutations, lesions of epigenetic modifiers and tumor suppressors were detected^11–13^. Only scattered data are available on the molecular features underlying CLPD-NK, mainly provided by approaches targeted to exons of specific genes, with limited discovery power. *STAT3* mutations were almost all identified in exons 20–21 encoding the Src homology 2 domain, with different prevalence in different cohorts^7,14–16^. Albeit relatively rare, *STAT3* mutations are regarded as a diagnostic tool, in addition to the aberrant NK-cell immunophenotype and bone marrow or splenic infiltration by cytotoxic lymphocytes^14^. CLPD-NK patients with *STAT3* mutations often require treatment and *STAT3* mutations have been found to correlate with a CD3− CD16+ CD56^dim^ CD57− immunophenotype^16^ and cytopenias, such as anemia^15^ or severe neutropenia^16^. *STAT5B*, a gene essential for NK cell biology^17^ and frequently mutated in CD4+ T-LGL leukemia^18^, has been found mutated only in one patient diagnosed with CLPD-NK (3%), who later progressed to ANKL^9^. Somatic mutations in few other genes of the JAK/STAT pathway, such as *JAK3* (10%)^14^, and of the NF-kappa B signaling pathway, such as *TNFAIP3* (6%)^15^, were seldom detected in CLPD-NK.

The fact that *STAT3* mutations were found only in a minority of CLPD-NK patients, and the observation of STAT3 activation and increased expression of genes activated by STAT3 also in patients with wild-type *STAT3*^7^ encouraged investigation of the CLPD-NK genetic profile of patients resulting negative after screening for *STAT3* and *STAT5B* mutations. CLPD-NK positioning relatively to T-LGLL, ANKL, and NKTCL, is unclear and CLPD-NK maintained a provisional status in the last 2017 WHO classification. Large studies informing CLPD-NK molecular profiling are still lacking and, in this regard, CLPD-NK remains a poorly characterized disease. Key open questions include CLPD-NK etiopathogenesis and molecular features underlying patient heterogeneity and possibly indicating targetable lesions.

In the present study of a sizeable CLPD-NK patient cohort, a low prevalence of *STAT3* mutations was confirmed and no *STAT5B* mutations were detected, prompting a deep WES analysis of the largest so far group of patients with CLPD-NK. All the considered patients had wild-type *STAT3* and *STAT5B* at screening. We disclosed still unexplored aspects of the molecular landscape of CLPD‐NK, finding mutations of genes wired to cancer proliferation, survival, and migration pathways and potential driver genes for which FDA-approved drugs are available.

## Material and methods

### Patients

A cohort of 57 CLPD-NK patients was recruited at the Hematology Unit of Padua University Hospital. The study and blood sample collection were approved by the Ethic Committee for Clinical Trials of Padua. The diagnosis followed WHO criteria (LGL count over 0.5 × 10^9^/L persisting for at least six months and CD3− CD16+ CD8± CD57± CD56± phenotype). Six of the patients considered in the study received treatment. Analyzed DNA was collected at diagnosis. Clinical data, including sex, age, presence of cytopenia and mutational status on *STAT3*/*STAT5B* genes were collected for each patient. According to the Helsinki Declaration, patients gave written informed consensus prior to inclusion in the study.

### *STAT3* and *STAT5B* mutation screening

DNA from purified leukemic clones of 57 CLPD-NK patients was screened for mutations in hotspot regions of *STAT3* and *STAT5B* genes, by Sanger sequencing or by Amplification Refractory Mutation System (ARMS) polymerase chain reaction (PCR).

For Sanger sequencing, DNA was amplified with primers^9,10^ (Supplementary Table 1) covering exons 19–21 for *STAT3* and exon 16 for *STAT5B* genes. Purified PCR products were sequenced using dye terminator technology and ABI 3130 sequencer (Applied Biosystem); Sequencing results were analyzed with ChromasPro software.

ARMS PCR was performed using outer primers to amplify the target region of *STAT3* gene and inner primer pairs to selectively amplify the variant (Y640F or D661Y^7^) or the wild-type allele.

### WES profiling

Ten representative CLPD-NK patients (Table 1) were selected for WES according to their immunophenotype, clinical features, and absence of *STAT3*/*STAT5B* mutations, determined by Sanger sequencing (Supplementary Table 1).

**Table 1:** Clinical and molecular features of the 13 CLPD-NK patients analyzed by WES.

CLPD-NK Pat.	Sex	Age	WBC	ANC	Hb	PLT	ALC	Immunophenotype	KIR expression restricted	NKG2
76	M	45	6.4	2.5	147	300	4.96*	CD16+ CD56+ CD57+	158B	C
100	M	52	10	2.36	151	227	9.12*	CD16+ CD56+ CD57+	158E	A
115	M	79	7	2.2	144	163	3.76*	CD16+ CD56+ CD57+	158B	Negative
117	M	49	7.1	2.67	155	245	5.24*	CD16+ CD56+ CD57+	158B	A
165	M	43	3.16*	1.32*	153	60*	1.90	CD16+ CD56+ CD57−	Not expressed	A
187	M	73	5.16	1.94	159	198	8.89*	CD16+ CD56− CD57+	Not expressed	A
260	M	59	12.35*	1.31*	133	238	9.95*	CD16+ CD56+ CD57+	Not expressed	A
337	M	67	5.6	2.53	147	217	4.42*	CD16+ CD56+ CD57+	158A	A
448	M	59	5	3.98	88*	317	4.15*	CD16+ CD56+ CD57+	Not expressed	A
452	M	44	6.4	2.29	145	207	5.31*	CD16+ CD56− CD57+	158B	A
1253	F	65	4.1	0.01*	120*	157	3.35	CD16+ CD56− CD57+	NA	NA
1272	F	47	7.7	NA	NA	NA	NA	CD16+CD56− CD57+	NA	NA
1260	F	46	10.9	1.49*	114*	281	10.89*	CD16+ CD56− CD57+	NA	NA

All patients resulted wild type for *STAT3* and *STAT5B* (see Methods). *indicates values outside the normal range (WBC 3.5–11 × 10^9^/L; ANC 1.9–5.3 × 10^9^/L; Hb 125–169 g/L; PLT 110–330 × 10^9^/L; ALC 1.18–3.62 × 10^3^/μL). All patients presented restricted KIR expression (either presence of only one KIR or total absence of KIR expression) and most frequently a heterodimer of the A type NKG2 with CD94+. *WBC* white blood count, *ANC* absolute neutrophil count, *ALC* absolute lymphocyte count, *Hb* hemoglobin, *PLT* platelets, *M* male, *F* female, *NA* not available.

Mononuclear cells (MC) and granulocytes were isolated from peripheral blood (PB) of CLPD-NK patients using Lymphocytes Separation Media (Biowest). NK cells were purified from PBMC using magnetic Micro-Beads coated with monoclonal anti-human CD57, CD56, or CD16 antibodies (Miltenyi Biotec), according to the dominant leukemic population. At least 97% purity of both NK leukemic cells and granulocytes (control sample) was confirmed by flow cytometry analysis. Contamination of monocytes in the residual fraction resulted below 1% in all samples.

DNA was extracted using Puregene cell and tissue kit (Qiagen). WES (Illumina platform, Agilent SureSelect 60 Mbp kit with paired end reads) was performed starting from 1 µg of DNA from both tumor and control samples for each patient.

### Somatic variant detection and prioritization

The study cohort (Table 1) of 13 patients comprised ten newly sequenced and three previously profiled by WES and analyzed with a different pipeline^3^.

WES data of tumor-control matched samples were analyzed by using an in-house made pipeline based on Docker^19^ which automatically performs bioinformatics analysis, from reads alignment to somatic variant calling and annotation (Supplementary Fig. 1). Somatic single-nucleotide polymorphisms (SNPs) and insertions/deletions (indels) were detected with three different variant callers to increase the discovery power: MuTect^20^, MuTect2^21^, and Strelka2^22^. Only variants called in regions with coverage of at least ten reads were kept.

Somatic variants with population allele frequency >5% were discarded, considering gnomAD (Genome Aggregation Database, https://gnomad.broadinstitute.org) data of non-Finnish European and Finnish populations for the analysis of patients from Italy and Finland, respectively.

Next, known variants were associated with dbSNP (v. 151)^23^ or COSMIC (v. 84)^24^ identifiers and those annotated as benign or likely benign in ClinVar (updated on 23/07/2018) were discarded. Only variants with SnpEff^25^ predicted impact *HIGH* or *MODERATE* and with MetaSVM^26^ predicted impact “*deleterious*” were further considered.

### Mutated gene network analysis

R Graphite Bioconductor package (v.1.28.2)^27^ was used to convert KEGG^28^ and Reactome^29^ pathway topologies into pathway-derived mutated gene networks, using appropriate biology-driven rules to transform different types of direct and indirect relations between genes and gene products annotated in pathways (i.e., regulatory relations, participation to molecular complexes, and biosynthetic pathways, also with compound intermediates) into pairwise connections between genes. Pathways including at least one mutated gene were converted into networks and merged in order to build up a unique nonredundant KEGG-Reactome meta-network. The meta-network was integrated also with protein–protein interactions derived from Reactome FI^30^. Genes with normalized expression lower than 1 in Human Protein Atlas (https://www.proteinatlas.org/) were not considered, finally depicting 43 pairwise direct, indirect and predicted interactions between 44 genes. The possible driver role of mutated genes was evaluated considering prediction made by Cancer Genome Interpreter^31^ (www.cancergenomeinterpreter.org), variant allele frequency (VAF) and impact on protein and gene function. Visualization, optimization and annotation of the network were performed using Cytoscape v3.5.1^32^.

### Variant validation

DNA from both purified leukemic clone and autologous granulocytes was analyzed to confirm the presence of the tested mutation only in the tumor counterpart. Variants were confirmed by Sanger sequencing (VAF at least 0.2) or ARMS PCR. Custom primers (Supplementary Table 1) were designed by Primer3, using GRCh37.p13 as reference, analyzed with IDT OligoAnalyzer Tool and obtained from Sigma-Aldrich.

## Results

### *STAT3* and *STAT5B* mutations are rare in CLPD-NK patients

The collected study group of 57 CLPD-NK patients (Supplementary Table 2) included the 25 cases previously screened for *STAT3* exon 21 and *STAT5B* exon 16 mutations^16^. All patients were diagnosed for CLPD-NK at the Hematology Unit of Padua University Hospital, from 2000 to 2019, and underwent routine immunophenotyping and molecular characterization, based on PBMC samples. The median age at diagnosis was 68 years, with a prevalence of male patients (82%). Abnormal white blood count, absolute neutrophil count (ANC), or hemoglobin level were recorded in 14% of patients, whereas only 4% of them had low count of platelets. Immunophenotypic analysis revealed that CD16+ CD56+ CD57+ expression, with a restricted pattern of KIR expression and CD94+ NKG2A+ heterodimer, was the most frequent among the cohort. Six patients (10.5%) underwent treatment, mostly related to the development of autoimmune hemolytic anemia or symptomatic neutropenia.

**Fig. 1:**
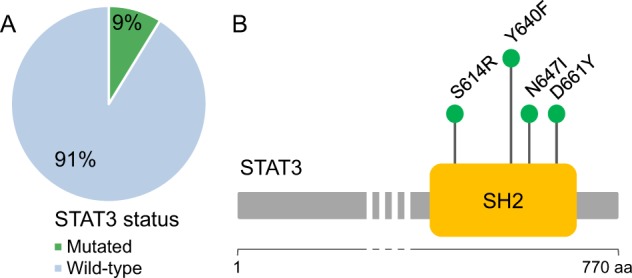
*STAT3* mutations in a cohort of 57 CLPD-NK patients. **a** *STAT3* was mutated in 5 out of 57 patients (9%). **b** Four different variants were observed in the SH2 domain.

*STAT3* mutation screening, in exons 19–21, detected somatic mutations in the SH2 domain only in 5 out of 57 patients studied (9%). Two cases carried Y640F mutation (detected by Sanger sequencing and ARMS PCR), whereas D661Y, N647I, and S614R mutations were found in one case each by Sanger sequencing (Fig. 1). Only two out of the six patients that required treatment carried *STAT3* mutations, presented neutropenia and CD3− CD16+ CD56^dim^ CD57− immunophenotype (Supplementary Table 2), in accordance with Barilà et al.^16^. The same immunophenotype was associated with a normal ANC in a third patient, whereas the last case had the most common CD3− CD16+ CD56^dim^ CD57+ immunophenotype.

None of the patients resulted mutated after screening of *STAT5B* exon 16, confirming that *STAT5B* mutations are rare in CLPD-NK.

### Deleterious somatic variants of the leukemic clone in CLPD-NK patients detected by WES profiling were validated

Among the 52 CLPD-NK patients negative for *STAT3/5B* mutations, ten with typical immunophenotype, representative of clinical manifestations (Table 1), including the two cases who required treatment, underwent WES profiling (Agilent SureSelect 60 Mbp, Illumina sequencing, paired end reads). For these cases, a highly purified leukemic clone and normal granulocytes, used as control, were used for WES.

After WES sequence read quality selection and alignment to the reference genome, a high and homogenous sequence coverage in the cohort was obtained (in average 159× and 142× for tumor and control samples, respectively; over 95% of target regions with coverage of at least 20; Supplementary Fig. 2). Variant calling with different methods (MuTect^20^, MuTect2^21^, and Strelka2^22^; Supplementary Fig. 1) detected 5124 SNPs and 119 indels with high confidence in the whole cohort. We excluded a significant correlation between the number of somatic variants and the mean coverage in tumor and in control samples (Supplementary Figure 3).

Next, variant annotation, filtering and analysis were conducted leveraging several software tools and databases (see the Methods section and Supplementary Figure 1). Briefly, known variants (in dbSNP and COSMIC) likely benign according to ClinVar, and/or with population allele frequency >5% (according to gnomAD) were discarded to focus on possible driver genes. To this aim, among the remaining variants, only those predicted to be “very deleterious” both according to SnpEff^25^ and MetaSVM^26^ were further considered. Thus, 867 variants (in 827 genes) rare in the reference population and also predicted to be deleterious by different methods were kept.

Somatic mutations with different VAF were detected in the leukemic samples (Supplementary Fig. 4). The VAF distribution was heterogeneous in different patients (Kruskal–Wallis test, *p* value = 2.2e−16), and overall 80% of variants had VAF lower than 0.05 in the NK leukemic cells; a very high (>97%) purity of these cells had been confirmed by flow cytometry analysis.

In addition, three CLPD-NK patients negative for *STAT3*/*STAT5B* mutations and previously profiled by WES^3^ were included in the study (Table 1). WES data of these patients were reanalyzed with the same computational pipeline and criteria used for newly sequenced samples.

**Fig. 2:**
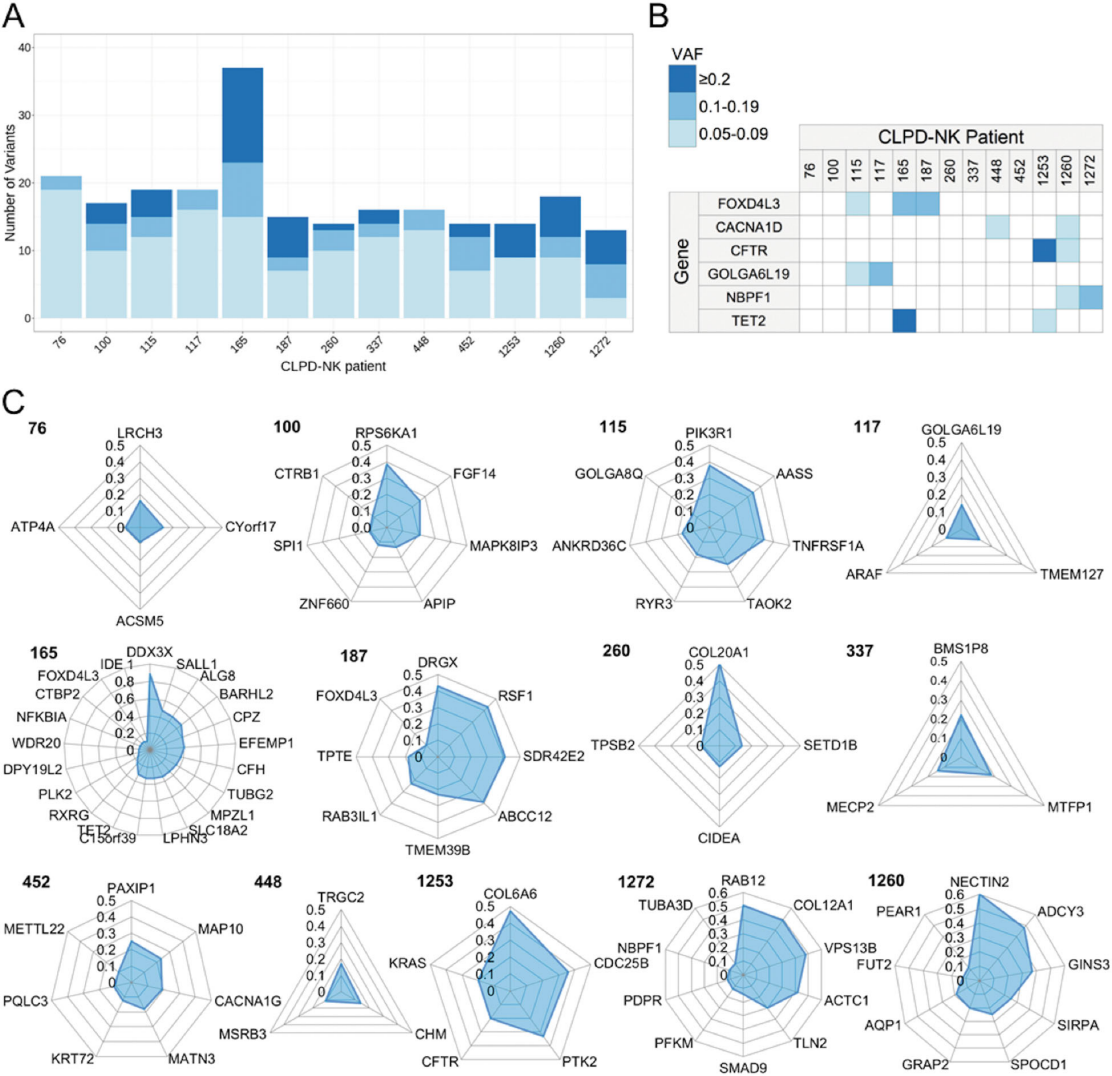
Heterogeneity of CLPD-NK patient leukemic clones disclosed multiple clonal and subclonal somatic mutations. **a** Number of mutations per patient and **b** summary of genes recurrently mutated in the cohort, showing different classes of VAF; **c** prominent genes in each patient are indicated by radar plots of VAF (only genes with somatic variants with VAF ≥ 0.1 are shown).

In the 13 CLPD-NK patients altogether, 235 deleterious somatic variants (217 SNPs and 18 indels, in 226 genes; Supplementary Table 3) present with VAF ≥ 0.05 were taken to further analysis, also considering the purity level. From 13 to 37 deleterious variants in different genes coexisted (Fig. 2a), with 6 genes recurrently mutated in the cohort (Fig. 2b). Figure 2c shows in more detail, for each patient, which and how many genes carried deleterious variants with VAF > 0.1, that we considered more likely to include the genes playing a role in the disease. Eighteen of these somatic variants with VAF > 0.1 were prioritized for validation according to variant type and particularly to gene function. Sanger sequencing or ARMS PCR, when the VAF was below 0.2, confirmed all the tested variants (Table 2, Supplementary Fig. 5). For several patients, validation of multiple co-occurring variants was obtained.

**Table 2:** Validated somatic variants in CLPD-NK patients.

CLPD-NK Pat.	Gene	Variant	VAF	Variant effect and involved domain
100	*FGF14*	p.Thr224Met	0.26	Missense variant close to an acetylation site
100	*MAPK8IP3*	p.Val1314fs	0.21	Deletion leading to stop codon in 1315 position
100	RPS6KA1*	p.Ser430fs	0.4	Frameshift variant in the PKinase domain
115	PIK3R1*	p.Ser565_Ile566insGluTyrArgGluIleAspLysArgMetAsnSer	0.38	In-frame insertion in protein-protein interaction site, predicted to be oncogenic by OncoKB
115	TAOK2*	p.Val244Met	0.25	Missense variant in the PKinase domain
115	TNFRSF1A*	p.Pro102Arg	0.34	Missense variant in the cysteine-rich domain important for ligand binding
117	*TMEM127*	p.His165Arg	0.12	Missense variant in the loop between transmembrane domains S3 and S4
165	*DDX3×*	c.104-2 A > G	0.89	Variant in splicing acceptor site of exon3
165	TET2*	p.Arg1465*	0.31	Truncating variant in the Oxygenase domain, likely oncogenic loss of function
165	RXRG*	c.623-1 G > C	0.22	Variant in splicing acceptor site of exon 4
165	*CFH*	p.Gly1118Glu	0.37	Missense variant in Sushi domain involved in immune recognition processes
165	*MPZL1*	p.Phe60Leu	0.34	Missense variant in V-set Immunoglobulin like domain
165	*EFEMP1*	p.Arg362*	0.40	Stop gain mutation in EGF like domain eliminating 5 phosphorylation sites
165	*SALL1*	p.Ala377fs	0.48	Frameshift mutation truncating the protein before all the functional domains
187	*RSF1*	p.Val68Met	0.43	Missense variant in the DDT domain involved in DNA binding
260	*SETD1B*	p.Ala889Asp	0.14	Missense variant possibly altering the secondary structure
452	*MAP10*	p.Leu846*	0.23	Truncating variant in HPHLAWLY domain
452	*PAXIP1*	p.Gly638Ser	0.25	Missense variant in the phospho-binding BRCT domain

For 18 somatic variants confirmed by Sanger sequencing or ARMS PCR, details about the variant type, VAF, and possible effects are provided; *indicate genes for which interactions with FDA-approved drug interactions are known (http://www.dgidb.org/).

### Mutations of genes of the JAK/STAT pathway are not common in CLPD-NK

In all the samples, WES profiling gave high coverage for *STAT3*, *STAT5B*, *JAK3*, and *TNFAIP3*, genes previously associated with the disease. Negativity for *STAT3* and *STAT5B* mutations in the 10 CLPD-NK patients, previously screened for mutations in the hotspot regions of these genes, was confirmed by WES, for all patients, including the two cases (#187 and 448) who required treatment afterwards. Patient #187 presented 6 different variants at high VAF; among these, *RSF1*, encoding a protein involved in the transcription of hepatitis B virus genes, was validated. On the contrary, patient #448 presented all the variants with a VAF lower than 0.2. Neither *JAK3*, found mutated in CLPD-NK by Kurt et al.^14^, nor other *JAK* genes, were mutated in our cohort.

Provided the reported prevalence and driving role of *STAT3* mutations^7^ in CLPD-NK and the previous findings of mutations of the STAT protein interactor *FLT3* in a T-LGLL patient without *STAT* lesions^3^, we searched for mutations in genes functionally related to JAK/STAT signaling in CLPD-NK patients, starting from STAT3 and STAT5B interactors in STRING database: only *PTK2/FAK1* was mutated in patient #1253 (VAF 0.35), as previously reported^3^. In addition, only one of the 162 genes annotated in the KEGG JAK/STAT pathway was mutated in the cohort: we confirmed in patient #115 a deleterious variant in *PIK3R1*, encoding the phosphoinositide-3-kinase regulatory subunit 1, predicted to be a driver loss of function mutation of a known tumor suppressor gene. PIK3R1 is indeed a pivotal kinase negatively regulating the catalytic subunit (*PIK3CA*) of the PI3K complex and antagonized by the tumor suppressor PTEN. PI3K enzymes, mediating interchange of phosphates on inositol phospholipid species at the plasma membrane, are responsible for the coordination of a range of cell functions, including proliferation and survival, and play a prominent role in natural killer cell biology^33^. Although *PIK3R1* participates in several connected signaling pathways, it is also downstream of JAK signaling, witnessing the interconnections between JAK/STAT and PI3K signaling^34^.

The duplication of 11 amino acids at position 565 in the coiled coil region of the protein was restricted to the leukemic clone, with VAF 0.38. This *PIK3R1* variant coexisted in patient #115 with mutations of other genes, all at a lower VAF (Fig. 2c). In summary, 2/13 (15%) *STAT* negative patients carried mutations in *STAT3* interactors or other genes annotated in the JAK/STAT pathway.

### Somatic mutations recurrent in CLPD-NK

Six genes were recurrently mutated in our cohort (Fig. 2b), five with only subclonal variants (VAF < 0.2). The transcription factor *FOXD4L3* was found mutated in three patients with VAF ranging from 0.09 to 0.12, whereas *CACNA1D, CFTR, GOLGA6L19*, and *NBPF1* carried subclonal variants in two CLPD-NK patients. *TET2* (Tet Methylcytosine Dioxygenase 2) tumor suppressor gene carried loss of function variants in two patients. The validated *TET2* variant (p.Arg1465*, VAF 0.31) in patient #165 abolished most of the oxygenase catalytic domain (aa 1290–1905). In patient #1253 the frameshift *TET2* p.Pro989fs variant (VAF 0.05) leads to a premature stop codon in 1008 position, before the catalytic domain. Deleterious somatic mutations in a large series of genes co-occurred with *TET2* mutation in patient #165, including a validated high VAF splicing variant of *DDX3X* gene encoding an ATPase/RNA helicase of the DEAD-box family, acting in RNA metabolism, cell cycle control, apoptosis, stress response and innate immunity.

We considered that deleterious variants in genes not expressed in NK cells may unlikely be regarded as disease drivers. Notably, *TET2* and *NBPF1* genes showed high expression in normal NK cells, according to the Human Protein Atlas^35^ (www.proteinatlas.org) RNA-seq data.

### Pathway-derived network of CLPD-NK somatic mutations

**Fig. 3:**
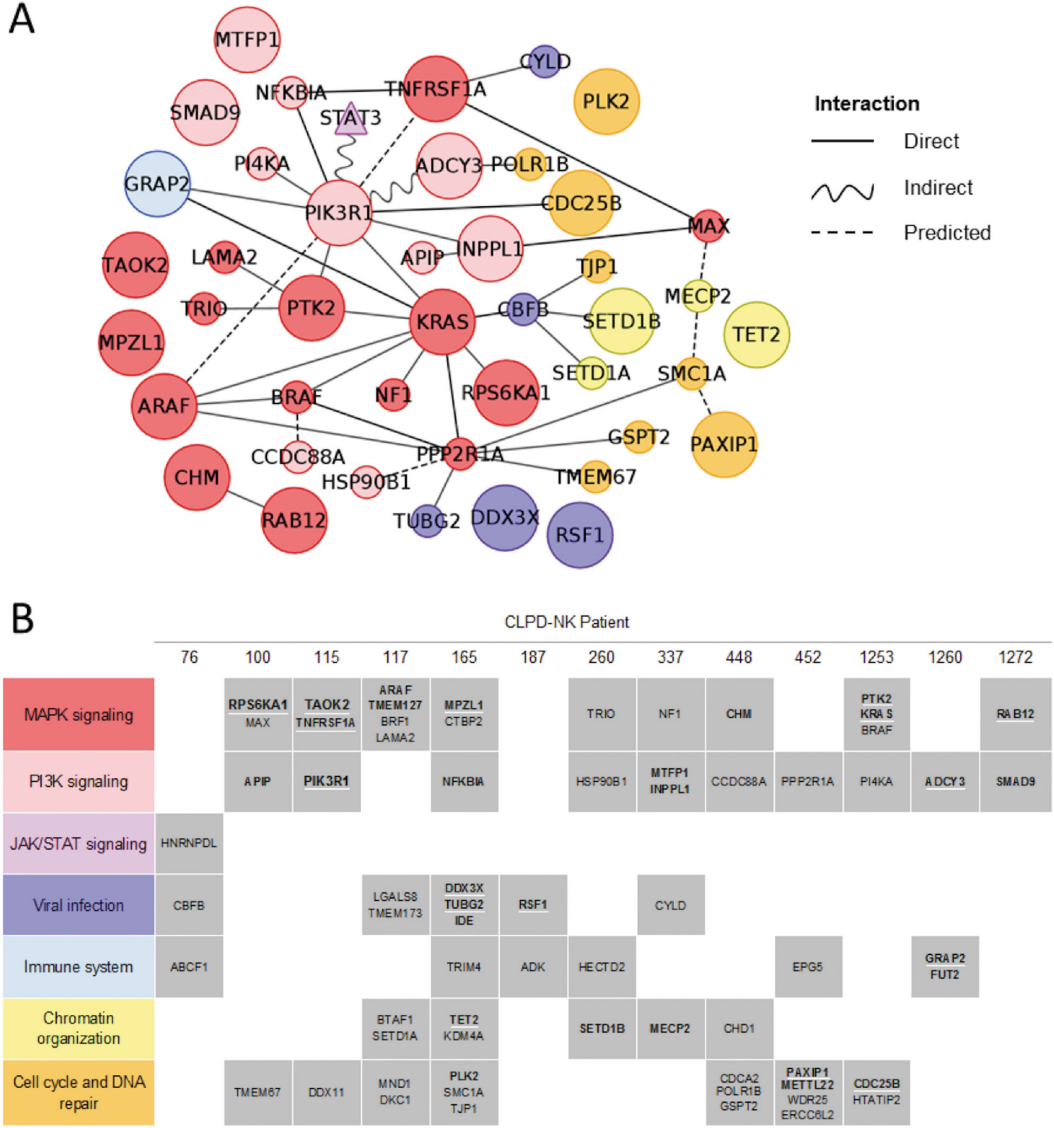
Summary of most relevant genes somatically mutated in CLPD-NK: functions and putative drivers. **a** KEGG and Reactome pathway-derived network of somatic mutations detected in CLPD-NK, by WES (circles) and by *STAT3* targeted screening (triangle); direct, indirect and predicted relations between genes and gene products annotated in pathways topologies are shown; node colors indicate different groups of pathways and functions, according to the table in panel (**b**); circular node size is proportional to the evaluation of possible driver role considering mutation VAF and predicted impact and gene function and expression; **b** Genes involved in different groups of pathways mutated in each patient highlight functions recurrently hit in different patients (gene name in bold, VAF ≥ 0.1; bold and underlined VAF ≥ 0.2).

Considering 109 genes expressed in NK cells and carrying deleterious variants, a data-driven picture of pathways and biological functions recurrently hit by somatic mutations in CLPD-NK patients emerged from the construction of an integrated mutation network based on KEGG and Reactome pathways and on protein–protein interactions (Fig. 3a). The 44 genes in the network, and several others aggregated in seven main biological functions as shown in Fig. 3b. The same figure summarizes also the most relevant findings for each patient, considering gene function and expression, mutation VAF, recurrence, impact on protein, and possible driver role evaluated by Cancer Genome Interpreter^31^.

Eleven CLPD-NK patients carried deleterious mutations in at least one of 28 genes participating to Ras/MAPK or PI3K-AKT signaling pathways, associated with cancer proliferation, survival, and migration (Fig. 3b). High VAF and very deleterious variants of several genes of these signalings (*PIK3R1*, *MPZL1*, *RPS6KA1*, *TAOK2*, and *TNFRSF1A)* were validated (Table 2) and were likely relevant considering the high expression of these genes. In addition, five patients carried also mutations in eight genes linked to viral infections, including the above mentioned tumor suppressor gene *DDX3X* that is frequently mutated in NKTCL^12^ and in ANKL^11^, and *RSF1* (Table 2). Other groups of functionally related genes hit in 10/13 (77%) patients comprised genes regulating cell cycle and DNA repair, such as *CDC25B*, *PLK2*, *PAXIP1*, and genes important for immune system functions and/or involved in Interleukin signaling (*GRAP2*). *TET2* and other epigenetic modulators were mutated in 5/13 patients (38%), including *SETD1B* whose variant was validated. Reanalysis of three patients previously studied^3^ with refined bioinformatic methods confirmed findings and uncovered a few additional deleterious variants of possible relevance (Supplementary Table 3), as *BRAF* and *TET2* mutations, coexisting at subclonal level with deleterious variants of *PTK2* and *KRAS* in patient #1253 and *NECTIN2* in patient #1260. In most patients, as confirmed by variant validations (Table 2), multiple mutations of putative functional importance were indeed present, often hitting the same functions (Fig. 3).

## Discussion

This study disclosed the molecular landscape of somatic mutations in CLPD‐NK by an exploration of the largest so far patient cohort analyzed by WES. Even though the relatively “benign” and indolent nature of the CLPD-NK disease is well recognized, the number and predicted impact of somatic variants and functions of mutated genes detected in this study indicated that the leukemic clone of CLPD-NK patients carried a heavy burden of lesions involving genes related to cancer proliferation, survival and migration. Co-existence in the leukemic clone of a few major deleterious mutations with subclonal lesions was also observed. Among clonal mutations, likely drivers were identified. These results considerably broaden our view of the genes and pathways frequently hit in CLPD-NK patients, opening new lines of investigation.

First, the examination of 57 CLPD-NK cases detected only five patients with mutations in the highly conserved SH2 domain mediating STAT3 protein dimerization and activation, triggering transcription of downstream genes. The low frequency of *STAT3* mutations in our cohort and the absence of *STAT5B* mutations in the 57 patients screened in the hotspot regions of these genes was in line with the complete absence in the cohort of 13 CLPD-NK analyzed by WES of *STAT3* and *STAT5B* mutations, even outside the regions covered by the screening. Collectively, these observations confirmed that the inclusion of patients negative for *STAT3* and *STAT5B* mutations enhanced the study discovery power.

Our cohort of 57 patients was characterized by a 9% prevalence of *STAT3* mutations, resulting lower in comparison with the 30% prevalence described on 50 CLPD-NK patients by Jerez et al.^7^. Even if the clinical diagnosis and inclusion criteria for CLPD-NK^36^ patients in the two cohorts were homogeneous, a lower proportion (14% vs. 60% from Jerez et al.^7^) of symptomatic patients in our cohort can possibly explain the molecular differences that have been observed, considering that a relation between the development of symptoms (mostly related to neutropenia) and the presence of *STAT3* mutations has been reported^16^.

A previously unreported CLPD-NK leukemic clone heterogeneity emerged thanks to the high sequencing depth and the use of variant calling methods suitable to detect low VAF somatic mutations. Despite the stringent variant filtering used, an average of 87 deleterious and population rare somatic variants per patient were present. Most (80%) of these variants were highly subclonal (VAF < 0.05) and could be in part due to clonal hematopoiesis, relatively common in elderly people^37^. Higher VAF mutations, more deeply investigated here, corresponded to 217 SNPs and 18 indels, in 226 genes, in the whole cohort. Mutations with VAF > 0.2 were present in almost all patients: in 15 different genes in patient #165 and from 1 to 7 different genes in other 11 patients. Only in patient #117 the three most prominent variants had all a relatively low VAF (0.14–0.1), with a missense variant of the oncogene *ARAF* being the most prominent. Of the two patients who required treatment, #187 presented variants with VAF at least 0.2 in 6 genes, including the chromatin regulator *RSF1* that carried a validated deleterious missense variant in the DNA-binding domain (Table 2), whereas patient #448 had only subclonal variants.

Notwithstanding the relatively high number of somatic variants in the CLPD-NK cohort, only six genes were mutated in at least two patients and for four of them a primary drivers role was not supported by the relatively low VAF and expression. Function, expression, previous evidence of involvement in hematological malignancies and variant VAF indicated *TET2* as the most notable mutated gene. Two patients carried truncating mutations that eliminate the C-terminal Oxygenase catalytic domain of *TET2*, an epigenetic and transcriptional regulator involved in DNA demethylation critical for immune homeostasis^35^ (Fig. 3b). *TET2* alterations were associated with development of myeloid^38^ and lymphoid^39^ malignancies, and a *TET2* truncating variant was observed in one ANKL case^11^. The validated loss-of-function *TET2* truncating variant was associated with a particularly high mutation burden in patient #165. The relatively young age of the patient (45 years) argues against clonal hematopoiesis as an explanation for such a high VAF *TET2* mutation present in the leukemic clone^37^. Importantly, in light of recent evidence of the role of *TET2* in maintenance of genome stability^35^, the high mutation burden observed by WES in patient #165, backed up by validation of deleterious variants of 6 genes (*DDX3X*, *EFEMP1*, *MPZL1*, *CFH*, *RXRG*, and *SALL1*), supports the driver role of *TET2* variant.

The pathogenetic relevance of JAK/STAT pathway mutations in T-LGLL is well established^3,9,10^ with a markedly high prevalence in patients. JAK/STAT mutations or amplifications were shown to be involved in ANKL^11^ and NKTCL^12^ as well, although with a lower prevalence as compared to T-LGLL. Genes of the JAK/STAT pathway reported to be associated to CLPD-NK (*STAT3*, *STAT5B*, and *JAK3*)^14,15^ were not mutated in the 13 patients analyzed by WES. Mutations of *TNFAIP3*^15^, previously found in CLPD-NK, were not detected in our cohort. Anyhow, deleterious variants in STAT interactors or genes annotated in the JAK/STAT pathway (*PTK2/FAK1* and *PIK3R1*) were present in two patients (15%) and can be regarded as primary drivers in these cases (Fig. 3). *PIK3R1* recurrent mutation in lymphoid malignancies^40^, with two mutational hotspot regions observed, and severe risk of transformation in BCR-ABL models by *PIK3R1* deletion^41^ directly implicate *PIK3R1* hyperactivation in leukemogenesis. The *PIK3R1* p.Ser565_Ile566insGluTyrArgGluIleAspLysArgMetAsnSer variant falls in a protein region previously shown to be at the interface with the C2 domain of catalytic subunit *PIK3CA*. Mutations in this region (e.g., aa 567) abrogate the negative regulation on *PIK3CA*, thereby promoting cell survival, Akt activation and oncogenesis^42,43^. *PIK3R1* is also involved in the regulation of apoptosis, as other genes carrying deleterious variants confirmed in this study: *TNFRSF1A* (Tumor necrosis factor receptor superfamily member 1A), encoding a subunit of a receptor whose activation triggers Caspase-8 and apoptosis also involved in Interleukin and TNF signaling, carried a validated p.Pro102Arg variant (VAF 0.34) in the second TNFR domain of the extracellular portion of the protein; *TAOK2* (TAO Kinase 2), an effector of caspase 8 involved in apoptosis induction, in DNA damage response and stress-activated MAPK cascade, carried a p.Val244Met variant (VAF 0.25) in the kinase domain (Table 2). Of note, three putative driver mutations (in *PIK3R1*, *TAOK2*, and *TNFRSF1A)* at comparable VAF were identified and validated in patient #115.

Key clues of the possible relevance of deleterious somatic mutations in CLPD-NK proteins were obtained leveraging systems genetics approaches. The pathway and protein-protein interaction network of CLPD-NK somatic mutations (Fig. 3a) was reconstructed to obviate the low recurrence at gene level and to discover if different but functionally connected genes were hit by mutations in different patients or by mutations co-occurring in the same clone. A group of 28 genes mutated in all CLPD-NK belonged to Ras/MAPK and PI3K-AKT signaling pathways, highly interconnected and associated with cancer proliferation, survival, and migration.

Collectively, our analysis of mutation recurrence, VAF and effect, and of gene function, involvement in pathways and interactions and expression, identified a restricted number of genes with a putative driver role, whose variants were amongst those validated. In a personalized and precision medicine perspective, a search in the Drug Gene Interaction Database, identified that for at least six of the genes with validated variants (*RPS6KA1*, *PIK3R1*, *TAOK2*, *TNFRSF1A*, and *TET2*) one or more FDA-approved drugs are already available (Table 2). For instance, cyclophosphamide interacts with *TNFRSF1A* and is already used in clinical management of T-LGLL as first line of treatment^44^, whereas hypomethylating agents (azacitidine and decitabine) are used to counteract *TET2* loss of function, in myeloproliferative disorders^45^ and the CDK inhibitor purvalanol A was shown to interact with *RPS6KA1*^46^.

Finally, our results informed on similarities and differences between CLPD-NK and other LGL lymphoproliferative diseases. Comparing the genetic landscapes of CLPD-NK and T-LGLL (near CLPD-NK in the 2017 WHO classification of mature T- and NK-cell neoplasms), possibly due to the limited size of both cohorts, a very limited overlap of genes and pathways was found. Ras/MAPK involvement^3^, however, common in several different malignancies, emerged in both neoplasms. By comparing diseases of mature NK cells, similarities emerged between the indolent CLPD-NK and the remarkably more aggressive ANKL and NKTCL. Commonly mutated genes had often a likely a driver role in CLPD-NK. *TET2* truncating variants were detected in CLPD-NK (2/13) and ANKL (1/14) and different SET Domain containing epigenetic modifiers were mutated in both diseases (*SETD1A* and *SETD1B* in CLPD-NK, *SETD2* in ANKL). *DDX3X* mutation, recurrent in ANKL^11^ and in NKTCL^47^, was observed here for the first time in CLPD-NK. Other genes mutated both in CLPD-NK and ANKL patients included *DDX11*, *RSF1*, and *KRAS*. Tyrosine phosphatase mutations were reported in ANKL and are considered a hallmark of NKTCL, whereas in our CLPD-NK cohort only one tyrosine phosphatase (*PTPRN*) was mutated. In ANKL and NKTCL a high Epstein Barr Virus (EBV) burden was previously detected^11^ by WES data analysis by Centrifuge^48^. At variance, our analysis performed with the same approach did not detect any EBV load in CLPD-NK patients (data not shown).

Our data confirmed a possible involvement in CLPD-NK of the JAK/STAT pathway, even though not directly since somatic mutations hit genes whose primary function is implicated in other signaling pathways. Of novelty, we showed that somatic mutations of CLPD-NK patient leukemic clones affect genes of the Ras/MAPK and PI3K pathways involved in cancer proliferation, survival and migration, or cell cycle, DNA repair and epigenetic regulator genes. This study broadened our knowledge of the CLPD-NK leukemic clone genetic profile in patients negative for *STAT3/5B* mutations, giving relevant new insights into the pathobiology of CLPD-NK and opening new possibilities for personalized treatment of patients.

## Supplementary information

Supplementary Figures

Supplementary Table 1

Supplementary Table 2

Supplementary Table 3

## Data Availability

WES data will be available upon request.
