# Supplementary material for: A high definition picture of somatic mutations in chronic lymphoproliferative disorder of natural killer cells
Source: Blood Cancer J. 2020 Apr 22;10(4):42. doi: 10.1038/s41408-020-0309-2 (PMC7176632; doi:10.1038/s41408-020-0309-2)
Supplement: Supplementary file 1 — Supplementary Figures [file 41408_2020_309_MOESM1_ESM.pdf]

A high definition picture of somatic mutations in Chronic Lymphoproliferative Disorder of Natural Killer cells

Vanessa Rebecca Gasparini[1,2]* Andrea Binatti[3]*, Alessandro Coppe[4,5], Antonella Teramo[1,2], Cristina Vicenzetto[1,2], Giulia Calabretto[1,2], Gregorio Barilà[1,2], Annica Barizza[1,2], Edoardo Giussani[3], Monica Facco[1,2], Satu Mustjoki[6,7], Gianpietro Semenzato[1,2$], Renato Zambello[1,2&] and Stefania Bortoluzzi[3,8&]

[1] Department of Medicine, Hematology and Clinical Immunology Branch, University of Padova, Padova, Italy;
[2] Veneto Institute of Molecular Medicine (VIMM), Padova, Italy;
[3] Department of Molecular Medicine, University of Padova, Padova, Italy;
[4] Department of Women's and Children's Health, University of Padova, Padova, Italy;
[5] Department of Biology, University of Padova, Padova, Italy;
[6] Hematology Research Unit Helsinki, Helsinki University Hospital Comprehensive Cancer Center, Helsinki, Finland;
[7] Translational Immunology Research Program and Department of Clinical Chemistry and Hematology, University of Helsinki, Helsinki, Finland;
[8] CRIBI Biotechnology Centre, University of Padova, Padova, Italy.
* Co-first author
[$] Corresponding author
[&] Co-last author

Supplementary Result

Supplementary Figure 1. Bioinformatics pipeline workflow. **2**

Supplementary Figure 2. Sequence coverage profile of the 10 CLPD-NK patients newly profiled by WES. **3**

Supplementary Figure 3. Dot plot of the number of called somatic variants per patient and sample mean coverage **4**

Supplementary Figure 4. Variant allele frequency (VAF) profiles of the 10 CLPD-NK patients of the Italian cohort analyzed by WES. **5**

Supplementary Figure 5. Examples of variant confirmation by Sanger Sequencing or ARMS PCR **6**

Supplementary Table 1. Primers used for *STAT3* and *STAT5B* mutation screening and for validation of somatic variants detected by WES. **7**

Supplementary Table 2. Clinical and molecular features of the cohort of 57 CLPD-NK patients. **7**

Supplementary Table 3. List of somatic variants in 13 CLPD-NK cases. **7**

Supplementary Figure 1. Bioinformatics pipeline workflow.
Reads mapping, reads processing, variant calling and annotation were automated by using SCons software building tool in a Docker container. The annotated variants were prioritized considering information from various databases and VAF. Only variants expressed in NK cells (http://www.proteinatlas.org) were retained in order to build the pathway-derived meta-network.

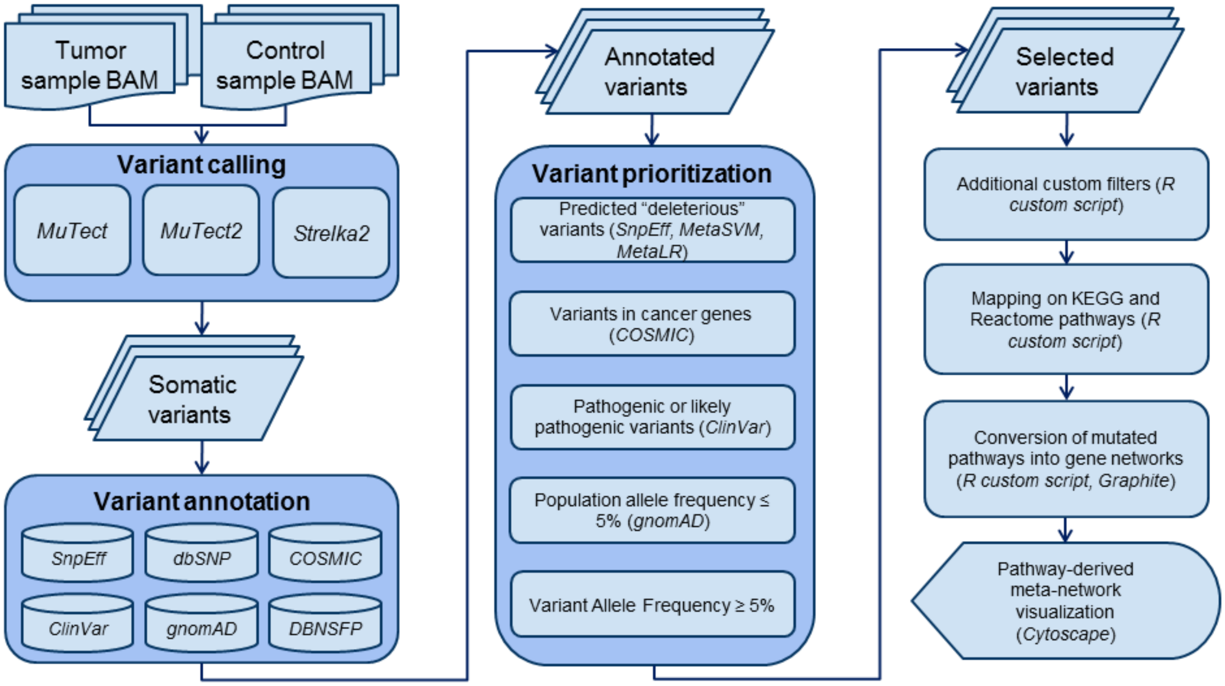

Supplementary Figure 2. Sequence coverage profile of the 10 CLPD-NK patients newly profiled by WES.

Bars indicate for each sample the percentage of target bases sequenced with different values of coverage; for each patient two bars are provided, corresponding to the tumor (T) and the control (N) samples.

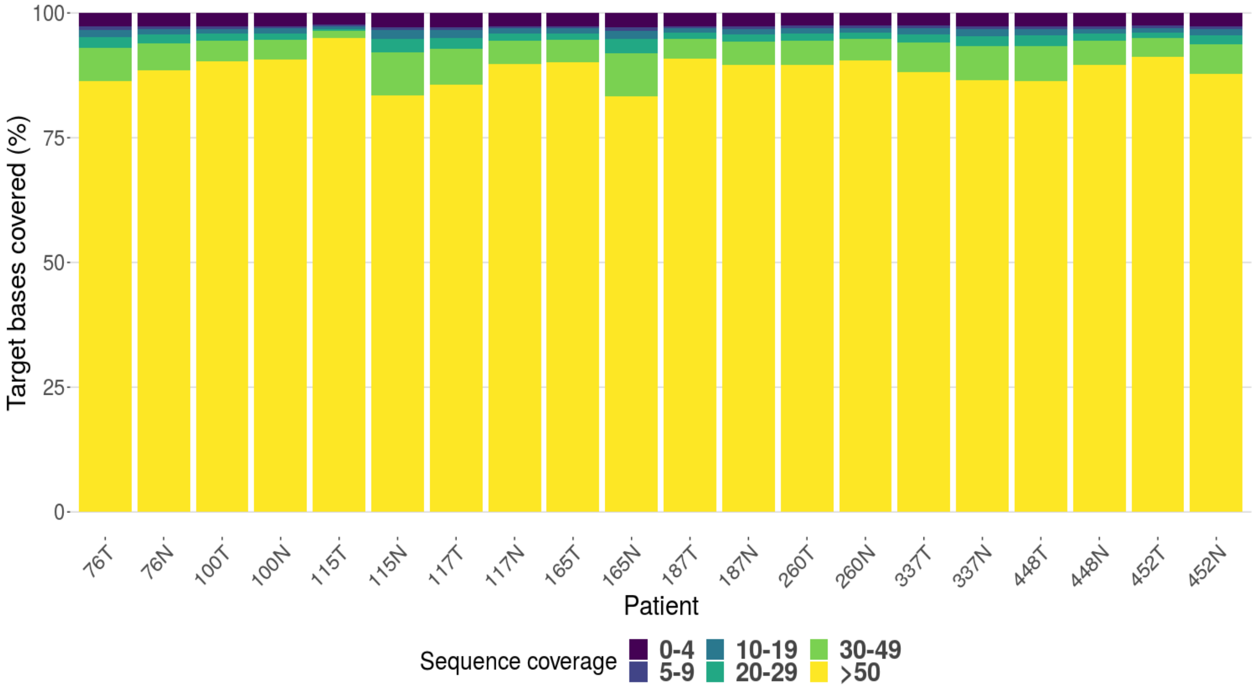

Supplementary Figure 3. Dot plot of the number of called somatic variants per patient and sample mean coverage.

Base-10 logarithm of the number of called somatic variants (y-axis) does not significantly correlate with the mean sequence coverage (A) in tumor samples (Spearman's test, rho = -0.15, p-value = 0.68) and (B) in control samples (Spearman's test, rho = 0.16, p-value = 0.66).

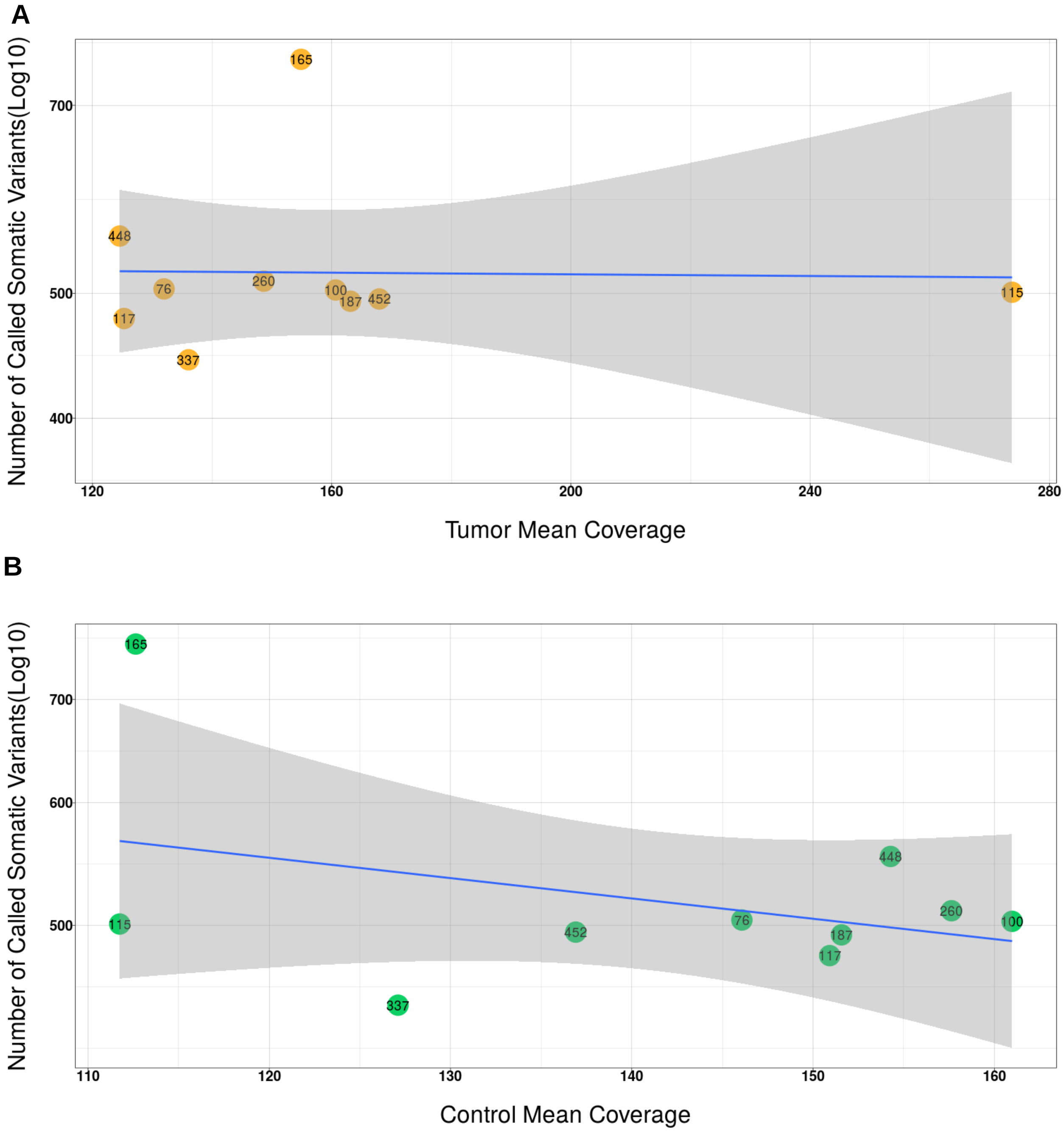

Supplementary Figure 4. Variant allele frequency (VAF) profiles of the 10 CLPD-NK patients of the Italian cohort analyzed by WES.

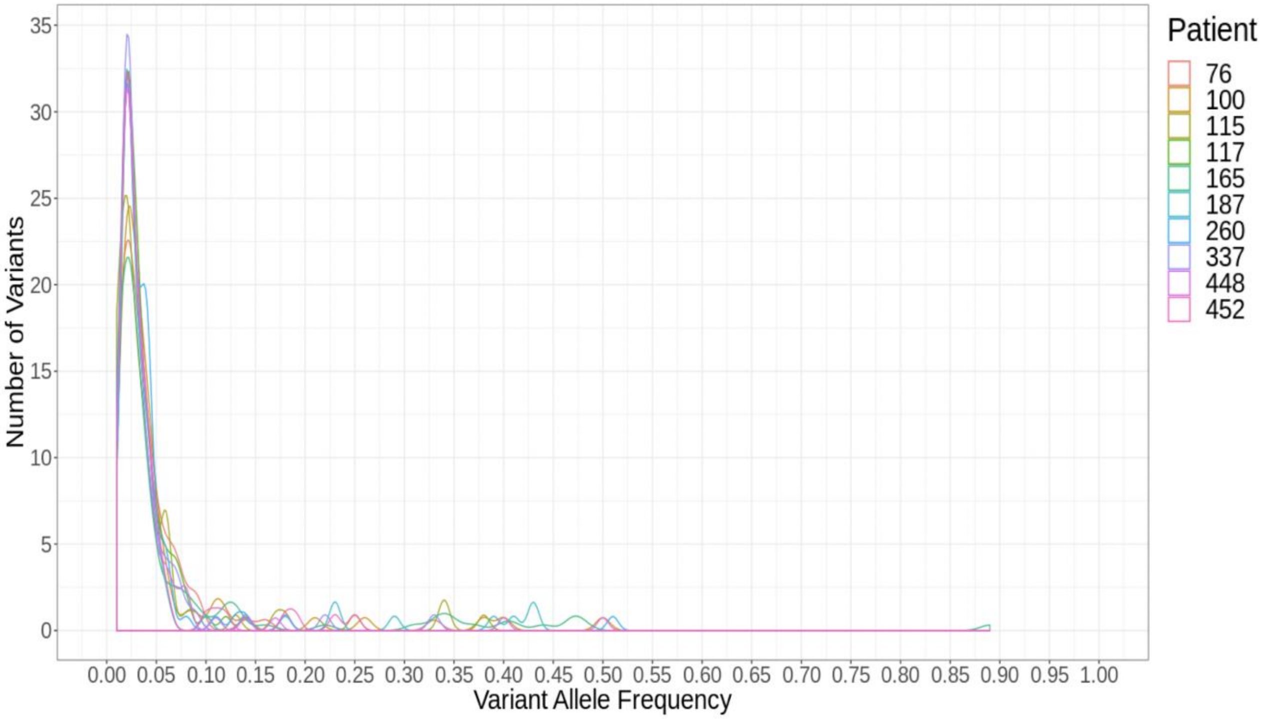

Supplementary Figure 5. Examples of variant confirmation by Sanger Sequencing or ARMS PCR.

A) Validation with Sanger sequencing of *TET2* truncating variant in patient 165: chromatograms of *TET2* wild type (wt) sequence in control sample and of *TET2* variant in the tumor sample are shown (left and right plots refer to the forward DNA strand and the reverse complement of the other strand, respectively); B) Validation with ARMS PCR of TMEM127 variant in patient 117 (product size: outer primers, 351bp; inner variant specific primers, 217bp; inner wt primers, 174bp).

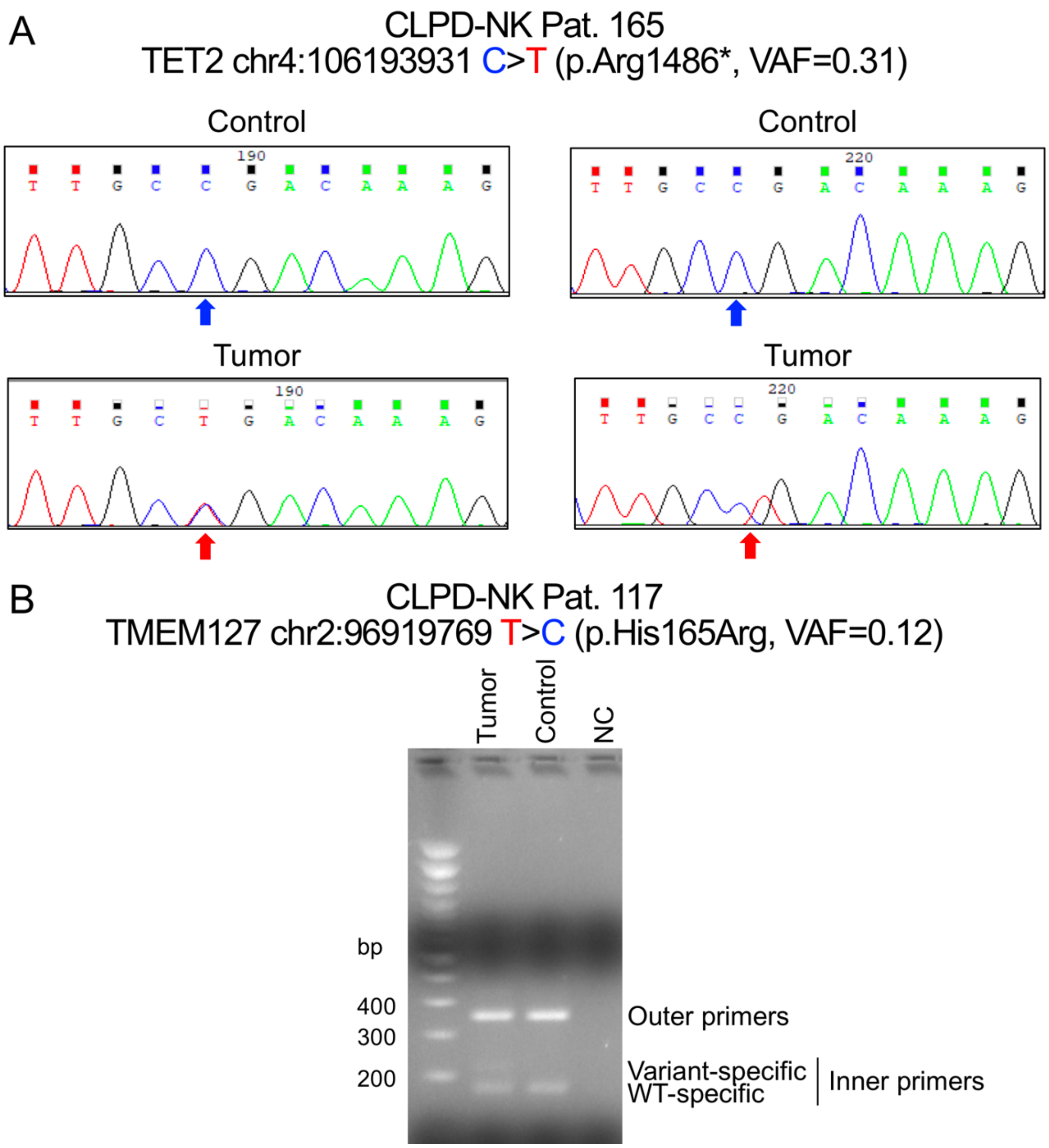

Supplementary Table 1. Primers used for *STAT3* and *STAT5B* mutation screening and for validation of somatic variants detected by WES.

(see separate .pdf file)

Supplementary Table 2. Clinical and molecular features of the cohort of 57 CLPD-NK patients.

(see separate .pdf file)

Supplementary Table 3. List of somatic variants in 13 CLPD-NK cases.

(see separate .pdf file)
