# Supplementary material for: A high definition picture of somatic mutations in chronic lymphoproliferative disorder of natural killer cells
Source: Blood Cancer J. 2020 Apr 22;10(4):42. doi: 10.1038/s41408-020-0309-2 (PMC7176632; doi:10.1038/s41408-020-0309-2)
Supplement: Supplementary file 2 — Supplementary Table 1 [file 41408_2020_309_MOESM2_ESM.pdf]

A high definition picture of somatic mutations in Chronic Lymphoproliferative Disorder of Natural Killer cells

Vanessa Rebecca Gasparini[1,2]* Andrea Binatti[3]*, Alessandro Coppe[4,5], Antonella Teramo[1,2], Cristina Vicenzetto[1,2], Giulia Calabretto[1,2], Gregorio Barilà[1,2], Annica Barizza[1,2], Edoardo Giussani[3], Monica Facco[1,2], Satu Mustjoki[6,7], Gianpietro Semenzato[1,2$], Renato Zambello[1,2&] and Stefania Bortoluzzi[3,8&]

[1] Department of Medicine, Hematology and Clinical Immunology Branch, University of Padova, Padova, Italy;
[2] Veneto Institute of Molecular Medicine (VIMM), Padova, Italy;
[3] Department of Molecular Medicine, University of Padova, Padova, Italy;
[4] Department of Women's and Children's Health, University of Padova, Padova, Italy;
[5] Department of Biology, University of Padova, Padova, Italy;
[6] Hematology Research Unit Helsinki, Helsinki University Hospital Comprehensive Cancer Center, Helsinki, Finland;
[7] Translational Immunology Research Program and Department of Clinical Chemistry and Hematology, University of Helsinki, Helsinki, Finland;
[8] CRIBI Biotechnology Centre, University of Padova, Padova, Italy.
* Co-first author
[$] Corresponding author
[&] Co-last author

Supplementary Table 1. Primers used for *STAT3* and *STAT5B* mutation screening and for validation of somatic variants detected by WES.

For each gene and variant, the primer pair used to amplify and sequence the genomic region is shown; for variants validated using ARMS PCR, two primer pairs are indicated, outer primers used to amplify the region and inner primers to selectively amplify the wild-type (wt) and the mutated allele.

Gene	Target	Forward primer	Reverse primer
STAT3	Exons 19-20	5'-ATCTCCACCCACCAGGGGGC-3'	5'-AGGGAAGGGCTGGGATGGCA-3'
	Exon 21	5'-TCCCATCGGTCACCCCAACA-3'	5'-GCCAGGCCACTGAACAGGGTG-3'
STAT5b	Exon 16	5'-TGTTGGGGTTTTAAGATTTCC-3'	5'-CAAATCAGAATGCGAACATTG-3'
CFH	p.Gly1118Glu	5’-CAGTGCTGTGTTTGCGTTTG-3’	5’-CACCCAGCCCTAAAGAGAAA-3’
DDX3X	splicing variant chrX:41200735 A->G	5'-GTGGAGGCTTTCAAATTCAGAA-3'	5'-ACAGGTGGTCAGGATAACGAC-3'
EFEMP1	p.Arg362*	5’-TGCTCTCACACCTCCTTCCT-3’	5’-CATGGCATTTGAGACTGGGC-3’
FGF14	p.Thr224Met	5'-TCCCATCACTAGTTGCCATGT-3'	5'-CCACGGAGCAGGAATGTCT-3'
MAP10	p.Leu846*	5'-TCTGGGAGGAAATGTGGAAATG-3'	5'-GTATGAGTGTACAGGTGACCC-3'
MAPK8IP3	p.Val1314fs	5'-GGAGGGCTACATCGACTTCC-3'	5'-AGAGGTTAGAAGAGGGGCGG-3'
MPZL1	p.Phe60Leu	5’-GCTGTATCTTTTCTTGTCTGGCA-3’	5’-GGATTTGATGGTGGAGAGGAAC-3’
PAXIP1	p.Gly638Ser	5'-TGTTCTTCTCTTGTATCCTGGGA-3'	5'-AGGCTATTTTCCTTACCTGTGC-3'
PIK3R1	p.Ser565_Ile566insGlu TyrArgGluIleAspLysArg MetAsnSer	5'-ACCTAAGGAAAACTGCTGGGA-3'	5'-ACTCGTTCAACTTCTTTTGCCG-3'
RPS6KA1	p.Ser430fs	5'-GGATGACCCCTAGCACTCTA-3'	5'-CGGCCACACACCTTTCATACA-3'
RXRG	splicing variant chr1:165380347 C->G	5’-CCCCTCATTCTCCTTAGGCA-3’	5’-GAGCACATACCGAGTTCTCCA-3’
RSF1	p.Val68Met	5'-ACACTGTCTGTTTTGAGAAGGT-3'	5'-TGGATTTTGGAGCCATAGTCTT-3'
SALL1	p.Ala377fs	5’-TACCACCCCGTCCTCTGAAA-3’	5’-TCGCTTCAAAGGCAGTGACA-3’
SETD1B	p.Ala889Asp	OUTER_5’-GCCACCACTGCCCAAGTTTG-3’	OUTER_5’-TAGTGGCTGCTGAAGTGGAG-3’
		INNER_VAR_SPEC-5’-GCAAGATGGTGGAAGTGGTAGA-3’	INNER_WT-5’-CTCGTCAAAGGCCCGGACAG-3’
TAOK2	p.Val244Met	5'-AGGTCATGTGGCAAGGAAGT-3'	5'-TCTGAGGTTGGTCTGTCTTGAG-3'
TET2	p.Arg1465*	5'-ACCAGGTATGCACTCTCACT-3'	5'-TGATGGGGCTGACTTTTCCT-3'
TMEM127	p.His165Arg	OUTER_5’-GGCAGGTTCTCTGAGAATG-3’	OUTER_5’-TCTTCCATCTCTGAGAGCAG-3’
		INNER_WT_5’-GCAGCAGCATAAGAAGTATCA-3’	INNER_VAR_SPEC-5’-GACATAGACCTGGGATCTAC-3’

TNFRSF1A	p.Pro102Arg	5'-GATGCAGGGACAGGAGGATG-3'	5'-ACAGGACAGAGGAAGTGACG-3'
