# Supplementary material for: A high definition picture of somatic mutations in chronic lymphoproliferative disorder of natural killer cells
Source: Blood Cancer J. 2020 Apr 22;10(4):42. doi: 10.1038/s41408-020-0309-2 (PMC7176632; doi:10.1038/s41408-020-0309-2)
Supplement: Supplementary file 3 — Supplementary Table 2 [file 41408_2020_309_MOESM3_ESM.pdf]

A high definition picture of somatic mutations in Chronic Lymphoproliferative Disorder of Natural Killer cells

Vanessa Rebecca Gasparini[1,2]* Andrea Binatti[3]*, Alessandro Coppe[4,5], Antonella Teramo[1,2], Cristina Vicenzetto[1,2], Giulia Calabretto[1,2], Gregorio Barilà[1,2], Annica Barizza[1,2], Edoardo Giussani[3], Monica Facco[1,2], Satu Mustjoki[6,7], Gianpietro Semenzato[1,2$], Renato Zambello[1,2&] and Stefania Bortoluzzi[3,8&]

[1] Department of Medicine, Hematology and Clinical Immunology Branch, University of Padova, Padova, Italy;
[2] Veneto Institute of Molecular Medicine (VIMM), Padova, Italy;
[3] Department of Molecular Medicine, University of Padova, Padova, Italy;
[4] Department of Women's and Children's Health, University of Padova, Padova, Italy;
[5] Department of Biology, University of Padova, Padova, Italy;
[6] Hematology Research Unit Helsinki, Helsinki University Hospital Comprehensive Cancer Center, Helsinki, Finland;
[7] Translational Immunology Research Program and Department of Clinical Chemistry and Hematology, University of Helsinki, Helsinki, Finland;
[8] CRIBI Biotechnology Centre, University of Padova, Padova, Italy.
* Co-first author
[$] Corresponding author
[&] Co-last author

Supplementary Table 2. Clinical and molecular features of the cohort of 57 CLPD-NK patients.

The asterisk indicates values outside the normal range (WBC 3.5-11x10^9/L; ANC 1.9-5.3x10^9/L; Hb 125-169g/L; PLT 110-330x10^9/L). M=male; F=female; WBC=white blood count; ANC=absolute neutrophil count; Hb=hemoglobin; PLT=platelets; For patients with restricted KIR expression, the table indicates the detected KIR type or the total absence. Regarding NKG2 receptor expression, activatory (A), inhibitory types (C), or absence are indicated; NA=not available; wt=wildtype; Targeted=Sanger sequencing of *STAT3* exon 21 and *STAT5B* exon 16; WES=Whole Exome Sequencing.

CLPD-NK Patient	Sex	Age	WBC	ANC	Hb	PLT	Treatment	Immunophenotype	KIR Expression	NKG2 Expression	STAT3 Status	STAT5B Status	Profiling
7	M	80	10.43	4.53	142	278	NO	CD16+ CD56+ CD57-	Normal	NA	wt	wt	Targeted
9	M	57	9.7	na	na	na	NO	CD16+ CD56+ CD57+	Absent	A	wt	wt	Targeted
14	M	77	5.43	3.15	161	195	NO	CD16+ CD56+ CD57+	Normal	NA	wt	wt	Targeted
15	M	50	6.06	2.5	137	229	NO	CD16+ CD56+ CD57+	Normal	Normal	wt	wt	Targeted
19	F	70	8.7	1.5	146	222	NO	CD16+ CD56+ CD57+	Absent	Negative	wt	wt	Targeted
22	M	79	5.86	2.86	141	195	NO	CD16+ CD56+ CD57+	Normal	Normal	wt	wt	Targeted
24	F	88	na	na	na	na	NO	NA	NA	NA	wt	wt	Targeted
38	M	88	10.55	4.35	160	197	NO	NA	NA	NA	wt	wt	Targeted
39	M	73	8.18	4.31	131	219	NO	CD16+ CD56+ CD57+	NA	A	wt	wt	Targeted
45	M	85	7.57	2.98	102*	219	NO	CD16+ CD56+ CD57+	158B	A	N647I	wt	Targeted
62	M	54	5.7	1.2*	153	414*	NO	CD16+ CD56+ CD57+	158E	A	wt	wt	Targeted
66	M	77	6.61	3.61	144	196	NO	CD16+ CD56+ CD57+	158B+158E	A	wt	wt	Targeted

76	M	45	6.4	2.5	147	300	NO	CD16+ CD56+ CD57+	158B	C	wt	wt	WES
90	M	65	7.1	3.3 3	162	242	NO	CD16+ CD56+ CD57+	Normal	Normal	wt	wt	Targeted
100	M	52	10	2.3 6	151	227	NO	CD16+ CD56+ CD57+	158E	A	wt	wt	WES
103	M	84	8.0 8	4.4 9	160	163	NO	CD16+ CD56+ CD57+	Normal	A	wt	wt	Targeted
115	M	79	7	2.2	144	163	NO	CD16+ CD56+ CD57+	158B	Negative	wt	wt	WES
117	M	49	7.1	2.6 7	155	245	NO	CD16+ CD56+ CD57+	158B	A	wt	wt	WES
133	M	76	3.4 8*	1.5 8	155	139	NO	CD16+ CD56+ CD57+	Absent	C	wt	wt	Targeted
151	F	83	3.7 5	1.9 6	148	142	NO	CD16+ CD56+ CD57-	Normal	Normal	Y640F	wt	Targeted
153	M	89	4.8 9	1.9 1	130	140	NO	CD16+ CD56+ CD57-	NA	NA	wt	wt	Targeted
164	M	56	8.5	2.7 7	168	153	NO	CD16+ CD56+ CD57+	158B	A	wt	wt	Targeted
165	M	43	3.1 6*	1.3 2*	153	60*	NO	CD16+ CD56+ CD57-	Absent	A	wt	wt	WES
175	F	79	1.8 5*	1.4 4*	84*	123	YE S	CD16+ CD56+ CD57+	Normal	Normal	wt	wt	Targeted
187	M	73	5.1 6	1.9 4	159	198	YE S	CD16+ CD56- CD57+	Absent	A	wt	wt	WES
194	M	83	7.8 8	3.2 4	151	245	NO	CD16+ CD56+ CD57+	Normal	Normal	wt	wt	Targeted
200	M	33	6.9	3.7 3	158	147	NO	CD16+ CD56+ CD57+	Absent	A	wt	wt	Targeted
216	M	80	8.1 9	4.5 8	162	249	NO	CD16+ CD56+ CD57-	Absent	C	wt	wt	Targeted
221	M	70	10	4.0 1	157	307	NO	CD16+ CD56+ CD57+	Absent	C	wt	wt	Targeted
228	M	82	2.9 2*	1.2 6*	96*	166	YE S	CD16+ CD56+ CD57-	Absent	A	D661Y	wt	Targeted

229	M	88	12.24*	5.69*	145	190	NO	CD16+ CD56+ CD57+	Absent	A	wt	wt	Targeted
241	M	71	6.24	na	na	na	NO	CD16+ CD56+ CD57-	Absent	A	wt	wt	Targeted
249	M	68	5.3	1.71	154	174	NO	CD16+ CD56+ CD57+	Normal	Normal	wt	wt	Targeted
252	F	54	5.27	3.77	149	189	NO	CD16+ CD56+ CD57-	Normal	Normal	wt	wt	Targeted
260	M	59	12.35*	1.31*	133	238	NO	CD16+ CD56+ CD57+	Absent	A	wt	wt	WES
266	F	74	12.63*	10.78*	139	188	YES	CD16+ CD56+ CD57+	Normal	A	wt	wt	Targeted
268	M	49	11	na	na	na	NO	CD16+ CD56+ CD57-	Normal	Normal	wt	wt	Targeted
285	F	63	4.06	2.1	147	216	NO	CD16+ CD56+ CD57-	Normal	Normal	wt	wt	Targeted
286	M	81	8.8	4.27	156	na	NO	CD16+ CD56+ CD57+	Normal	Normal	wt	wt	Targeted
289	M	80	2.73*	0.94*	138	250	NO	CD16+ CD56+ CD57+	Normal	Normal	wt	wt	Targeted
303	M	71	9.9	3.6	121*	394*	NO	CD16+ CD56+ CD57+	158B	A	wt	wt	Targeted
304	M	90	12.9*	na	na	na	NO	CD16+ CD56+ CD57+	158B	C	wt	wt	Targeted
312	M	84	4.9	na	na	na	NO	CD16+ CD56+ CD57-	Absent	A	S614R	wt	Targeted
316	F	70	3.5	0.6*	117*	130	YES	CD16+ CD56+ CD57-	Absent	A	Y640F	wt	Targeted
322	F	48	2.6*	0.35*	132	225	NO	CD16+ CD56+ CD57-	Normal	Normal	wt	wt	Targeted
337	M	67	5.6	2.53	147	217	NO	CD16+ CD56+ CD57+	158A	A	wt	wt	WES
339	M	68	9.94	6.37*	121*	223	NO	CD16+ CD56+ CD57+	158B	A	wt	wt	Targeted
343	F	57	12.4*	na	na	na	NO	CD16+ CD56+ CD57+	Absent	A	wt	wt	Targeted

351	F	42	14. 9*	na	na	na	NO	CD16+ CD56+ CD57-	Absent	A	wt	wt	Targeted
352	M	49	6.3 4	2.2 3	161	265	NO	CD16+ CD56+ CD57-	158A	A	wt	wt	Targeted
353	M	84	na	na	na	na	NO	CD16+ CD56+ CD57-	Normal	Normal	wt	wt	Targeted
394	M	83	na	na	na	70*	NO	CD16+ CD56- CD57+	Normal	A	wt	wt	Targeted
396	M	66	4.8	na	na	na	NO	CD16+ CD56- CD57+	Normal	A	wt	wt	Targeted
412	M	48	3.4 *	3.7	na	na	NO	CD16+ CD56+ CD57+	NA	A	wt	wt	Targeted
448	M	59	5	3.9 8	88*	317	YE S	CD16+ CD56+ CD57+	Absent	A	wt	wt	WES
452	M	44	6.4	2.2 9	145	207	NO	CD16+ CD56- CD57+	158B	A	wt	wt	WES
465	M	57	6.8	2.6	156	187	NO	CD16+ CD56+ CD57+	158B	Normal	wt	wt	Targeted
