# Supplementary material for: A high definition picture of somatic mutations in chronic lymphoproliferative disorder of natural killer cells
Source: Blood Cancer J. 2020 Apr 22;10(4):42. doi: 10.1038/s41408-020-0309-2 (PMC7176632; doi:10.1038/s41408-020-0309-2)
Supplement: Supplementary file 4 — Supplementary Table 3 [file 41408_2020_309_MOESM4_ESM.pdf]

A high definition picture of somatic mutations in Chronic Lymphoproliferative Disorder of Natural Killer cells

Vanessa Rebecca Gasparini[1,2]* Andrea Binatti[3]*, Alessandro Coppe[4,5], Antonella Teramo[1,2], Cristina Vicenzetto[1,2], Giulia Calabretto[1,2], Gregorio Barilà[1,2], Annica Barizza[1,2], Edoardo Giussani[3], Monica Facco[1,2], Satu Mustjoki[6,7], Gianpietro Semenzato[1,2$], Renato Zambello[1,2&] and Stefania Bortoluzzi[3,8&]

[1] Department of Medicine, Hematology and Clinical Immunology Branch, University of Padova, Padova, Italy;
[2] Veneto Institute of Molecular Medicine (VIMM), Padova, Italy;
[3] Department of Molecular Medicine, University of Padova, Padova, Italy;
[4] Department of Women's and Children's Health, University of Padova, Padova, Italy;
[5] Department of Biology, University of Padova, Padova, Italy;
[6] Hematology Research Unit Helsinki, Helsinki University Hospital Comprehensive Cancer Center, Helsinki, Finland;
[7] Translational Immunology Research Program and Department of Clinical Chemistry and Hematology, University of Helsinki, Helsinki, Finland;
[8] CRIBI Biotechnology Centre, University of Padova, Padova, Italy.
* Co-first author
[$] Corresponding author
[&] Co-last author

Supplementary Table 3. List of somatic variants in 13 CLPD-NK cases.

For each variant information about the impact scoring on involved transcripts (H, High; M, Moderate), amino acid change and variant allele frequency (VAF) are provided. Ref=reference; Alt=Alternative.

Chr	Position	Ref.	Alt.	Gene	Transcript	Impact	AA change	Patient	VAF
1	120301845	C	T	HMGCS2	ENST00000369406, ENST00000544913	H, M	p.Glu249Gly, p.Glu207Gly	76	0.06
1	1372840	T	C	VWA1	ENST00000476993	H	p.Gln203*	100	0.05
1	156879548	T	C	PEAR1	ENST00000292357, ENST00000338302	H, H	p.Gln473*, p.Gln473*	1260	0.12
1	165380347	G	C	RXRG	ENST00000359842, ENST00000470566	H, H	NA, NA	165	0.22
1	167734908	G	C	MPZL1	ENST00000359523, ENST00000448405, ENST00000392121, ENST00000474859, ENST00000367853	M, M, M, M, M	p.Phe60Leu, p.Phe60Leu, p.Phe60Leu, p.Phe60Leu, p.Phe34Leu	165	0.34
1	16902915	A	G	NBPF1	ENST00000430580, ENST00000432949, ENST00000287968	M, M, M	p.Arg656Trp, p.Arg114Trp, p.Arg21Trp	1272	0.11
1	16902931	G	C	NBPF1	ENST00000430580, ENST00000432949, ENST00000287968	M, M, M	p.Gln650His, p.Gln108His, p.Gln15His	1260	0.08
1	16910159	C	T	NBPF1	ENST00000430580	M	p.Met341Val	1260	0.09
1	180124226	C	G	QSOX1	ENST00000367602, ENST00000367600	H, H	p.Ala62Pro, p.Ala62Pro	117	0.06
1	196714989	A	G	CFH	ENST00000367429	M	p.Gly1118Glu	165	0.37

1	199998337	A	G	NR5A2	ENST00000474307	M	p.Gly81Arg	165	0.44
1	228346067	A	G	GJC2	ENST00000366714	M	p.Arg203His	115	0.05
1	232943306	A	T	MAP10	ENST00000418460	H	p.Leu846*	452	0.23
1	236702250	A	G	LGALS8	ENST00000341872, ENST00000366584, ENST00000526634	H, H, H	p.Arg69His, p.Arg69His, p.Arg69His	117	0.07
1	23696043	T	A	C1orf213	ENST00000335648	H	p.Arg85*	1253	0.05
1	26887289	T	TC	RPS6KA1	ENST00000531382, ENST00000374168, ENST00000374166, ENST00000526792, ENST00000374162, ENST00000530003	H, H, H, H, H, H	p.Ser439fs, p.Ser430fs, p.Ser419fs, p.Ser338fs, p.Ser338fs, p.Ser414fs	100	0.38
1	32264081	A	G	SPOCD1	ENST00000360482, ENST00000257100, ENST00000452755, ENST00000533231, ENST00000528579	M, M, M, M, M	p.Arg664Cys, p.Arg157Cys, p.Arg101Cys, p.Arg664Cys, p.Arg79Cys	1260	0.25
1	32538637	C	CGGGGGGAG GAGATGGGT GAGCAGAGC GGCTCAGGC TCGGCCT	TMEM39B	ENST00000336294, ENST00000438825, ENST00000441402, ENST00000456834, ENST00000336294, ENST00000438825, ENST00000441402, ENST00000472503, ENST00000456834, ENST00000468135, ENST00000373634, ENST00000427288	H, H, H, H, H, H, H, H, H, H, H, H	p.Met1fs, p.Met1fs, p.Met1fs, p.Met1fs, NA, NA, NA, NA, NA, NA, NA, NA	187	0.23
1	44163657	A	C	KDM4A	ENST00000372396	M	p.Ser938Arg	165	0.05
1	6648246	T	C	ZBTB48	ENST00000466813	M	p.Ser94Phe	76	0.07

1	82408979	A	G	LPHN2	ENST00000370717, ENST00000370721, ENST00000370723, ENST00000370725, ENST00000370727, ENST00000370728, ENST00000370730, ENST00000359929, ENST00000319517, ENST00000370713, ENST00000370715, ENST00000271029, ENST00000335786, ENST00000394879, ENST00000449420	M, M, M, M, M, M, M, M, M, M, M, M, M, M, M	p.Glu242Lys, p.Glu246Lys, p.Glu242Lys, p.Glu242Lys, p.Glu242Lys, p.Glu242Lys, p.Glu242Lys, p.Glu242Lys, p.Glu242Lys, p.Glu242Lys, p.Glu242Lys, p.Glu242Lys, p.Glu242Lys, p.Glu242Lys, p.Glu109Lys	115	0.06
1	91178116	A	G	BARHL2	ENST00000370445	M	p.Ala306Val	165	0.47
1	9305319	A	G	H6PD	ENST00000602477, ENST00000377403	M, M	p.Arg120His, p.Arg109His	76	0.05
2	101180452	T	G	GOT1	ENST00000370508, ENST00000543866	H, M	p.Leu77Met, p.Leu56Met	260	0.05
2	119014839	T	C	SLC18A2	ENST00000298472	M	p.Pro251Leu	165	0.34
2	124266340	A	G	HTRA1	ENST00000368984, ENST00000420892	M, M	p.Gly304Asp, p.Gly45Asp	260	0.05
2	126683150	GT	AC	CTBP2	ENST00000309035, ENST00000337195, ENST00000334808, ENST00000531469, ENST00000494626, ENST00000411419	M, M, M, M, M, M	p.Val763Thr, p.Val223Thr, p.Val291Thr, p.Val223Thr, p.Val223Thr, p.Val223Thr	165	0.12
2	126683166	A	C	CTBP2	ENST00000309035, ENST00000337195, ENST00000334808, ENST00000531469, ENST00000494626, ENST00000411419	H, H, H, H, H, H	p.Glu758*, p.Glu218*, p.Glu286*, p.Glu218*, p.Glu218*, p.Glu218*	165	0.09
2	134594390	C	T	INPP5A	ENST00000368594, ENST00000445580	M, M	p.Ile376Thr, p.Ile58Thr	76	0.07

2	50574163	C	A	DRGX	ENST00000434016, ENST00000374139	H, H	p.Ter269Glyext *?, p.Ter264Glyext *?	187	0.43
2	73490361	T	G	CDH23	ENST00000224721	M	p.Gly1244Trp	76	0.07
2	76158290	T	G	ADK	ENST00000286621, ENST00000539909, ENST00000372734, ENST00000541550	H, H, H, H	p.Glu170*, p.Glu170*, p.Glu153*, p.Glu135*	187	0.05
2	93258812	T	C	HECTD2	ENST00000446394, ENST00000298068, ENST00000536715, ENST00000371667	M, M, M, M	p.Leu623Phe, p.Leu619Phe, p.Leu208Phe, p.Leu269Phe	260	0.06
2	93741960	T	C	BTAF1	ENST00000265990	M	p.Arg676Trp	117	0.07
2	94220947	A	C	IDE	ENST00000265986, ENST00000371581, ENST00000496903	H, H, H	NA, NA, NA	165	0.1
3	100179094	A	C	CNTN5	ENST00000279463, ENST00000527185, ENST00000528682, ENST00000524871, ENST00000418526	M, M, M, M, M	p.Pro875His, p.Pro875His, p.Pro875His, p.Pro875His, p.Pro801His	448	0.06
3	118272368	T	C	RP11-770J1.5	ENST00000534438	H	p.Trp5*	337	0.05
3	20398261	G	A	HTATIP2	ENST00000419348, ENST00000421577, ENST00000443524, ENST00000451739	H, H, H, H	p.Lys181Glu, p.Lys147Glu, p.Lys147Glu, p.Lys147Glu	1253	0.05
3	34904988	A	ATCTTT	APIP	ENST00000278359, ENST00000532428, ENST00000395787	H, H, H, H, H	p.Lys191fs, p.Lys127fs, p.Lys174fs	100	0.14
3	47380276	G	GCA	SPI1	ENST00000533968	H	p.Cys204fs	100	0.11

3	5529917	C	CTGG	UBQLN3	ENST00000311659	M	p.Thr290del	448	0.08
3	61674924	A	G	RAB3IL1	ENST00000394836, ENST00000301773, ENST00000531922	H, H, H	p.Arg127*, p.Arg174*, p.Arg174*	187	0.23
3	71936137	T	C	INPPL1	ENST00000298229	M	p.Arg37Trp	337	0.09
3	77475720	T	C	RSF1	ENST00000308488, ENST00000360355, ENST00000528095, ENST00000440064	M, M, M, M	p.Val68Met, p.Val68Met, p.Val67Met, p.Val65Met	187	0.43
3	77832226	C	T	ALG8	ENST00000526737	M	p.Met133Val	165	0.47
4	104331574	A	G	HSP90B1	ENST00000299767	H	p.Trp282*	260	0.05
4	121426681	T	G	HNF1A	ENST00000541395, ENST00000257555, ENST00000400024, ENST00000560968, ENST00000402929, ENST00000543427, ENST00000538646, ENST00000541924, ENST00000544413	M, M, M, M, M, M, M, M, M	p.Gln124His, p.Gln124His, p.Gln124His, p.Gln171His, p.Gln124His, p.Gln7His, p.Gln124His, p.Gln124His, p.Gln124His	100	0.05
4	122252787	A	C	SETD1B	ENST00000604567, ENST00000542440, ENST00000267197	M, M, M	p.Ala889Asp, p.Ala889Asp, p.Ala889Asp	260	0.14
4	25380282	C	G	KRAS	ENST00000256078, ENST00000311936	H, M	p.Ala59Gly, p.Ala59Gly	1253	0.2
4	31250830	G	C	DDX11	ENST00000407793, ENST00000542838, ENST00000228264, ENST00000545668, ENST00000350437	M, M, M, M, M	p.Gln592Glu, p.Gln592Glu, p.Gln566Glu, p.Gln592Glu, p.Gln592Glu	115	0.06
4	40876551	A	G	MUC19	ENST00000454784	M	p.Gly2699Glu	165	0.07

4	48531565	A	C	PFKM	ENST00000340802, ENST00000359794, ENST00000395233, ENST00000551804, ENST00000547587, ENST00000312352	M, M, M, M, M, M	p.Ala404Asp, p.Ala333Asp, p.Ala302Asp, p.Ala302Asp, p.Ala333Asp, p.Ala333Asp	1272	0.13
4	49959417	A	C	MCRS1	ENST00000548602	M	p.Trp4Leu	76	0.05
4	5154832	G	A	KCNA5	ENST00000252321	M	p.Thr507Ala	448	0.05
4	52986236	T	C	KRT72	ENST00000293745, ENST00000537672, ENST00000354310, ENST00000398066	M, M, M, M	p.Ala248Thr, p.Ala248Thr, p.Ala248Thr, p.Ala60Thr	452	0.12
4	54394210	C	T	HOXC9	ENST00000303450, ENST00000508190	M, M	p.Tyr80His, p.Tyr80His	115	0.06
4	56118211	A	G	RDH5	ENST00000257895, ENST00000547072, ENST00000548082	M, M, M	p.Arg280His, p.Arg183His, p.Arg280His	448	0.05
4	58218089	A	T	CTDSP2	ENST00000398073, ENST00000549039, ENST00000550144	M, M, M	p.Lys142Met, p.Lys24Met, p.Lys111Met	165	0.06
4	63984820	G	T	DPY19L2	ENST00000439061	H	p.Ter149Cysext *?	165	0.13
4	6442546	C	G	TNFRSF1 A	ENST00000534885	M	p.Pro102Arg	115	0.34
4	65720715	C	G	MSRB3	ENST00000355192, ENST00000308259, ENST00000540804, ENST00000535664, ENST00000541189, ENST00000538045, ENST00000535239, ENST00000446731	H, H, H, H, H, H, H, H	NA, NA, NA, NA, NA, NA, NA, NA	448	0.11

5	102375254	A	G	FGF14	ENST00000376131,<br>ENST00000376143	M, M	p.Thr229Met,<br>p.Thr224Met	100	0.26
5	25262530	T	C	ATP12A	ENST00000218548,<br>ENST00000381946	M, M	p.Thr101Met,<br>p.Thr101Met	165	0.07
5	37427796	T	G	SMAD9	ENST00000379826,<br>ENST00000399275,<br>ENST00000350148	H, H,<br>H	p.Tyr340*,<br>p.Tyr340*,<br>p.Tyr303*	1272	0.14
6	100848001	A	G	WDR25	ENST00000335290,<br>ENST00000554998,<br>ENST00000402312,<br>ENST00000554175,<br>ENST00000557710	M, M,<br>M, M,<br>M	p.Gly247Asp,<br>p.Gly247Asp,<br>p.Gly247Asp,<br>p.Gly247Asp,<br>p.Gly247Asp	452	0.06
6	102664675	T	G	WDR20	ENST00000558567	M	p.Trp156Cys	165	0.13
6	105692992	A	C	BRF1	ENST00000546474,<br>ENST00000392557,<br>ENST00000379937,<br>ENST00000446501,<br>ENST00000379932,<br>ENST00000551787,<br>ENST00000327359,<br>ENST00000440513,<br>ENST00000547562,<br>ENST00000549655,<br>ENST00000552127,<br>ENST00000546417	M, M,<br>M, M,<br>M, M,<br>M, M,<br>M, M,<br>M, M	p.Gln298His,<br>p.Gln94His,<br>p.Gln271His,<br>p.Gln60His,<br>p.Gln94His,<br>p.Gln94His,<br>p.Gln183His,<br>p.Gln183His,<br>p.Gln18His,<br>p.Gln94His,<br>p.Gln94His,<br>p.Gln151His	117	0.05
6	106092202	T	C	IGHG4	ENST00000390543	M	p.Val68Met	165	0.09
6	19560825	T	G	POTEG	ENST00000552966	M	p.Leu306Phe	448	0.06
6	35872513	A	AAGTGCCTC<br>AGCAATTTC<br>TGGCTGGTT<br>GGTGATCAC<br>AGCCAAGTG<br>GAGTGG	NFKBIA	ENST00000216797,<br>ENST00000554001,<br>ENST00000557140,<br>ENST00000557389	H, H,<br>H, H	p.Pro114fs,<br>p.Leu77fs,<br>p.Pro114fs,<br>p.Pro24fs	165	0.12

6	65560458	A	G	MAX	ENST00000358664, ENST00000341653, ENST00000358402, ENST00000394606, ENST00000284165, ENST00000553928, ENST00000556979, ENST00000555667, ENST00000557746, ENST00000556443, ENST00000246163	M, M, M, M, M, M, M, M, M, M, M	p.Arg47Trp, p.Arg47Trp, p.Arg38Trp, p.Arg47Trp, p.Arg47Trp, p.Arg47Trp, p.Arg47Trp, p.Arg38Trp, p.Arg38Trp, p.Arg38Trp, p.Arg47Trp	100	0.05
7	30019027	A	G	TJP1	ENST00000356107, ENST00000346128, ENST00000400011, ENST00000545208	H, H, H, H	p.Arg757*, p.Arg757*, p.Arg761*, p.Arg757*	165	0.07
7	30854100	C	T	GOLGA8Q	ENST00000562783	M	p.Ile492Thr	115	0.13
7	33952555	A	G	RYR3	ENST00000389232, ENST00000415757	M, M	p.Arg1518His, p.Arg1518His	115	0.18
7	35084618	A	C	ACTC1	ENST00000290378	M	p.Val203Phe	1272	0.42
7	63088416	A	G	TLN2	ENST00000306829, ENST00000561311, ENST00000494733	M, M, M	p.Asp1992Asn, p.Asp1992Asn, p.Asp906Asn	1272	0.3
7	75500267	A	AG	C15orf39	ENST00000360639, ENST00000394987, ENST00000567617, ENST00000565074	H, H, H, H	p.Gly627fs, p.Gly627fs, p.Gly627fs, p.Gly495fs	165	0.13
7	83013580	G	A	GOLGA6L19	ENST00000557886	M	p.Cys335Arg	117	0.14
7	83014053	T	C	GOLGA6L19	ENST00000557886	M	p.Gly177Glu	115	0.08
7	89876871	A	G	POLG	ENST00000268124, ENST00000442287	H, H	p.Gln39*, p.Gln39*	260	0.08

8	1031972	A	G	SOX8	ENST00000293894	M	p.Gly17Asp	165	0.05
8	1279908	CG	GC	TPSB2	ENST00000445910, ENST00000430512	M, M	p.Arg15Pro, p.Arg15Pro	260	0.11
8	1818752	A	AG	MAPK8IP3	ENST00000250894, ENST00000356010	H, H	p.Val1314fs, p.Val1308fs	100	0.21
8	20435328	A	G	ACSM5	ENST00000331849, ENST00000573920	H, H	p.Trp286*, p.Trp73*	76	0.09
8	22193543	T	C	SDR42E2	ENST00000602312	M	p.Ala240Val	187	0.41
8	28769318	T	G	NPIPB9	ENST00000550983	H	NA	115	0.05
8	29993056	A	G	TAOK2	ENST00000308893, ENST00000543033, ENST00000279394, ENST00000416441	M, M, M, M	p.Val244Met, p.Val244Met, p.Val244Met, p.Val71Met	115	0.25
8	30976417	T	G	SETD1A	ENST00000262519	H	p.Glu452*	117	0.05
8	33497584	C	T	BMS1P8	ENST00000565156	H	NA	337	0.22
8	48174597	T	C	ABCC12	ENST00000311303, ENST00000534418, ENST00000529084, ENST00000416054, ENST00000448542, ENST00000497206, ENST00000529504, ENST00000532494, ENST00000528693, ENST00000533639	H, H, H, H, H, H, H, H, H, H	NA, NA, NA, NA, NA, NA, NA, NA, NA, NA	187	0.39

8	50784037	T	C	CYLD	ENST00000311559, ENST00000569681, ENST00000569418, ENST00000540145, ENST00000564326, ENST00000566206, ENST00000566679, ENST00000398568, ENST00000427738, ENST00000568704	M, M, M, M, M, M, M, M, M, M	p.Pro143Leu, p.Pro143Leu, p.Pro143Leu, p.Pro143Leu, p.Pro143Leu, p.Pro143Leu, p.Pro143Leu, p.Pro143Leu, p.Pro143Leu, p.Pro143Leu	337	0.05
8	51175002	A	AG	SALL1	ENST00000251020, ENST00000440970, ENST00000570206	H, H, H	p.Ala377fs, p.Ala280fs, p.Ala280fs	165	0.48
8	58437189	G	A	GINS3	ENST00000318129	H	p.His125Arg	1260	0.37
8	67070558	C	G	CBFB	ENST00000412916, ENST00000290858	M, M	p.Gly61Ala, p.Gly61Ala	76	0.06
8	70154480	G	A	PDPR	ENST00000288050, ENST00000568530, ENST00000565186	M, M, M	p.Thr29Ala, p.Thr29Ala, p.Thr29Ala	1272	0.12
8	75258060	T	G	CTRB1	ENST00000361017	M	p.Gly202Trp	100	0.11
8	8739843	T	C	METTL22	ENST00000562151	M	p.Arg160Cys	452	0.1
8	87885434	T	C	SLC7A5	ENST00000261622	M	p.Cys187Tyr	260	0.06
9	29587481	T	C	NF1	ENST00000358273, ENST00000356175, ENST00000579081, ENST00000456735	M, M, M, M	p.Arg1509Cys, p.Arg1488Cys, p.Arg1522Cys, p.Arg1154Cys	337	0.05
9	34854201	A	C	MYO19	ENST00000431794, ENST00000268852	M, M	p.Cys889Phe, p.Cys689Phe	1253	0.07
9	36091619	A	G	HNF1B	ENST00000225893, ENST00000561193, ENST00000427275, ENST00000560016	H, H, H, H	p.Gln338*, p.Gln312*, p.Gln312*, p.Gln338*	1260	0.08

9	40818620	T	G	TUBG2	ENST00000251412, ENST00000588870	H, H	NA, NA	165	0.35
9	48655668	A	G	CACNA1G	ENST00000359106, ENST00000360761, ENST00000354983, ENST00000352832, ENST00000442258, ENST00000416767, ENST00000502264, ENST00000503436, ENST00000503485, ENST00000503607, ENST00000504076, ENST00000505165, ENST00000506406, ENST00000507336, ENST00000507510, ENST00000507609, ENST00000507896, ENST00000510115, ENST00000510366, ENST00000511765, ENST00000511768, ENST00000512389, ENST00000513689, ENST00000513964, ENST00000514079, ENST00000514181, ENST00000514717, ENST00000515165, ENST00000515411, ENST00000515765, ENST00000429973, ENST00000358244	M, M, M, M, M, M, M, M, M, M, M, M, M, M, M, M, M, M, M, M, M, M, M, M, M, M, M, M, M, M, M, M	p.Asp682Asn, p.Asp682Asn	452	0.19
9	58235127	T	C	CA4	ENST00000300900, ENST00000590203	M, M	p.Ala164Val, p.Ala12Val	448	0.07
9	74134048	C	CA	FOXJ1	ENST00000322957	H	p.Val218fs	100	0.09
10	12254540	A	G	CIDEA	ENST00000522713	M	p.Arg53His	260	0.13
10	211214	A	G	USP14	ENST00000261601, ENST00000582707, ENST00000400266, ENST00000383589	H, H, H, H	p.Trp472*, p.Trp437*, p.Trp461*, p.Trp426*	76	0.05

10	24128148	C	T	KCTD1	ENST00000417602	M	p.Glu118Gly	115	0.06
10	31325759	T	C	ASXL3	ENST00000269197	H	p.Gln1983*	1272	0.09
10	43479395	A	G	EPG5	ENST00000282041, ENST00000590884, ENST00000592272	H, H, H	p.Gln1595*, p.Gln470*, p.Gln470*	452	0.06
10	8638227	TT	GC	RAB12	ENST00000329286	M	p.Pro235Ser	1272	0.51
11	36051825	T	C	ATP4A	ENST00000262623	M	p.Ala144Thr	76	0.09
11	36831287	C	CATAAGGTT	ZFP14	ENST00000270001	H	p.Lys478fs	165	0.08
11	45377732	T	C	PVRL2	ENST00000252483, ENST00000252485, ENST00000591581	H, H, H	p.Arg347*, p.Arg347*, p.Arg187*	1260	0.6
11	49206407	T	C	FUT2	ENST00000391876, ENST00000522966, ENST00000425340	M, M, M	p.Thr65Met, p.Thr65Met, p.Thr65Met	1260	0.12
11	49935796	T	C	SLC17A7	ENST00000221485, ENST00000543531, ENST00000600601	M, M, M	p.Arg377His, p.Arg365His, p.Arg310His	117	0.05
11	52714556	T	G	PPP2R1A	ENST00000322088, ENST00000454220, ENST00000444322	H, M, M	p.Arg105Leu, p.Arg145Leu, p.Arg50Leu	452	0.05
12	11295812	A	C	PQLC3	ENST00000295083, ENST00000445402, ENST00000445921, ENST00000441908, ENST00000402361, ENST00000428481	M, M, M, M, M, M	p.Ala47Glu, p.Ala70Glu, p.Ala47Glu, p.Ala47Glu, p.Ala47Glu, p.Ala26Glu	452	0.11

12	113332590	T	C	POLR1B	ENST00000541869, ENST00000263331, ENST00000409894, ENST00000537335, ENST00000417433, ENST00000458012	M, M, M, M, M, M	p.Pro936Ser, p.Pro898Ser, p.Pro715Ser, p.Pro687Ser, p.Pro842Ser, p.Pro283Ser	448	0.08
12	132240243	T	A	TUBA3D	ENST00000321253	M	p.Asp392Val	1272	0.1
12	133483236	A	C	NCKAP5	ENST00000317721, ENST00000409261, ENST00000409213, ENST00000405974	H, H, H, H	p.Gly1893*, p.Gly1893*, p.Gly574*, p.Gly574*	1253	0.09
12	141816500	T	C	LRP1B	ENST00000389484	M	p.Glu454Lys	260	0.06
12	164466217	A	G	FIGN	ENST00000333129	M	p.Pro709Ser	100	0.05
12	189859505	T	G	COL3A1	ENST00000304636, ENST00000317840	M, M	p.Gly468Val, p.Gly468Val	1272	0.07
12	20205958	C	G	MATN3	ENST00000407540, ENST00000421259	M, M	p.Leu113Val, p.Leu113Val	452	0.18
12	202466564	C	T	ALS2CR11	ENST00000439140, ENST00000286195, ENST00000439802, ENST00000450242	M, M, M, M	p.Ile138Met, p.Ile138Met, p.Ile138Met, p.Ile138Met	337	0.07
12	220156202	T	C	PTPRN	ENST00000295718, ENST00000409251, ENST00000443981, ENST00000423636	H, M, M, M	p.Val907Met, p.Val878Met, p.Val109Met, p.Val817Met	1253	0.07
12	240954157	C	T	NDUFA10	ENST00000252711, ENST00000404554	M, M	p.Asp223Gly, p.Asp223Gly	187	0.05
12	25065146	T	G	ADCY3	ENST00000260600, ENST00000427849, ENST00000435135, ENST00000433852	H, H, H, H	p.Tyr311*, p.Tyr67*, p.Tyr261*, p.Tyr89*	1260	0.48

12	27731060	T	C	GCKR	ENST00000264717, ENST00000424318, ENST00000411584	M, M, M	p.Ser455Phe, p.Ser265Phe, p.Ser155Phe	187	0.05
12	50780054	C	G	NRXN1	ENST00000404971, ENST00000406316, ENST00000405472, ENST00000401669, ENST00000402717, ENST00000406859	M, M, M, M, M, M	p.Ala517Gly, p.Ala477Gly, p.Ala469Gly, p.Ala477Gly, p.Ala469Gly, p.Ala477Gly	452	0.05
12	55573319	A	T	CCDC88A	ENST00000436346, ENST00000336838, ENST00000263630, ENST00000413716	H, H, H, H	p.Arg345*, p.Arg345*, p.Arg345*, p.Arg345*	448	0.05
12	56098175	A	G	EFEMP1	ENST00000355426, ENST00000394555, ENST00000394554, ENST00000424836	H, H, H, H	p.Arg362*, p.Arg362*, p.Arg362*, p.Arg224*	165	0.4
12	70164151	A	G	MXD1	ENST00000409442	M	p.Asp65Asn	337	0.05
12	96519588	A	G	ANKRD36C	ENST00000456556, ENST00000420871, ENST00000419039	H, H, H	p.Gln1673*, p.Gln924*, p.Gln700*	115	0.17
12	96919769	C	T	TMEM127	ENST00000258439, ENST00000432959, ENST00000435268	M, M, M	p.His165Arg, p.His165Arg, p.His81Arg	117	0.12
13	1895796	CA	TG	SIRPA	ENST00000400068, ENST00000356025, ENST00000358771	M, M, M	p.Leu44Ser, p.Leu44Ser, p.Leu44Ser	1260	0.25
13	29624034	AG	A	FRG1B	ENST00000439954	H	p.Ile25fs	115	0.06
13	34295892	A	C	RBM39	ENST00000338163	M	p.Trp410Cys	115	0.06
13	3785550	A	G	CDC25B	ENST00000439880	H	p.Arg521His	1253	0.36

13	43036095	A	G	HNF4A	ENST00000316099, ENST00000316673, ENST00000609795, ENST00000457232, ENST00000609262, ENST00000443598, ENST00000415691	M, M, M, M, M, M, M	p.Arg122Gln, p.Arg100Gln, p.Arg100Gln, p.Arg100Gln, p.Arg97Gln, p.Arg122Gln, p.Arg122Gln	337	0.06
13	48604589	A	G	SNAI1	ENST00000244050	H	p.Arg246His	260	0.05
13	61936894	A	G	COL20A1	ENST00000326996, ENST00000435874, ENST00000358894, ENST00000422202	M, M, M, M	p.Ala107Thr, p.Ala107Thr, p.Ala107Thr, p.Ala107Thr	260	0.51
14	10916369	T	C	TPTE	ENST00000361285, ENST00000298232, ENST00000342420, ENST00000447568	H, H, H, H	NA, NA, NA, NA	187	0.18
15	21064209	T	TTGTC	PI4KA	ENST00000255882, ENST00000414196, ENST00000572273, ENST00000399213	H, H, H, H	p.Thr2053fs, p.Thr805fs, p.Thr1995fs, p.Thr386fs	1253	0.05
15	23264852	A	G	IGLC7	ENST00000390331	M	p.Val29Ile	117	0.07
15	30821929	G	A	MTFP1	ENST00000266263, ENST00000355143, ENST00000412752, ENST00000407550	M, M, M, M	p.Tyr21Cys, p.Tyr21Cys, p.Tyr21Cys, p.Tyr21Cys	337	0.18
15	40367084	A	G	GRAP2	ENST00000344138, ENST00000543252, ENST00000540310, ENST00000544756, ENST00000399090, ENST00000407075	M, M, M, M, M, M	p.Arg330Gln, p.Arg278Gln, p.Arg264Gln, p.Arg258Gln, p.Arg217Gln, p.Arg330Gln	1260	0.2
15	46318809	T	C	WNT7B	ENST00000409496, ENST00000339464, ENST00000410089, ENST00000339464	M, M, M, M	p.Cys330Tyr, p.Cys326Tyr, p.Cys310Tyr, NA	100	0.05

16	113878687	A	G	DRD3	ENST00000383673, ENST00000460779, ENST00000467632, ENST00000295881	M, M, M, M	p.Arg100Cys, p.Arg100Cys, p.Arg100Cys, p.Arg100Cys	187	0.05
16	130307950	A	G	COL6A6	ENST00000358511, ENST00000453409, ENST00000506143, ENST00000511332	M, M, M, M	p.Arg1381Lys, p.Arg1381Lys, p.Arg158Lys, p.Arg138Lys	1253	0.47
16	13679306	A	G	FBLN2	ENST00000404922, ENST00000535798, ENST00000295760, ENST00000492059	M, M, M, M	p.Val1195Met, p.Val1174Met, p.Val1148Met, p.Val1195Met	1253	0.08
16	197607465	A	C	LRCH3	ENST00000438796, ENST00000428136	M, M	p.Ala764Glu, p.Ala141Glu	76	0.16
16	38592323	T	C	SCN5A	ENST00000333535, ENST00000414099, ENST00000423572, ENST00000413689, ENST00000425664, ENST00000443581, ENST00000451551, ENST00000455624, ENST00000450102, ENST00000449557	H, M, M, M, M, M, M, M, M, M	p.Arg1847His, p.Arg1829His, p.Arg1846His, p.Arg1847His, p.Arg1829His, p.Arg1846His, p.Arg1793His, p.Arg1814His, p.Arg1793His, p.Arg1793His	337	0.06
16	4358778	T	C	SETMAR	ENST00000358065, ENST00000425863	H, H	p.Gln635*, p.Gln496*	187	0.05
16	44635716	T	A	ZNF660	ENST00000322734, ENST00000416644, ENST00000441021	H, H, H	p.Lys11*, p.Lys11*, p.Lys11*	100	0.12
16	53769408	A	G	CACNA1D	ENST00000288139, ENST00000350061, ENST00000422281, ENST00000481478	M, M, M, M	p.Val897Ile, p.Val877Ile, p.Val877Ile, p.Val570Ile	448	0.07

16	53835150	T	G	CACNA1D	ENST00000288139, ENST00000350061, ENST00000422281, ENST00000481478, ENST00000544977	M, M, M, M, M	p.Gln1722His, p.Gln1702His, p.Gln1687His, p.Gln1395His, p.Gln81His	1260	0.06
16	86010720	T	C	CADM2	ENST00000405615, ENST00000383699, ENST00000407528	M, M, M	p.Thr291Met, p.Thr298Met, p.Thr289Met	115	0.06
17	106158062	AG	A	TET2	ENST00000513237, ENST00000305737, ENST00000540549, ENST00000545826, ENST00000394764, ENST00000265149, ENST00000380013, ENST00000413648	H, H, H, H, H, H, H, H	p.Pro1010fs, p.Pro989fs, p.Pro989fs, p.Pro989fs, p.Pro989fs, p.Pro989fs, p.Pro989fs, p.Pro989fs	1253	0.05
17	106193931	T	C	TET2	ENST00000513237, ENST00000540549, ENST00000380013	H, H, H	p.Arg1486*, p.Arg1465*, p.Arg1465*	165	0.31
17	154335928	A	G	MND1	ENST00000240488	H	p.Trp179*	117	0.08
17	156784688	T	A	ASIC5	ENST00000537611	M	p.Tyr87Asn	165	0.07
17	46252558	A	C	GABRA2	ENST00000507069, ENST00000510861, ENST00000514090, ENST00000381620, ENST00000356504, ENST00000540012	M, M, M, M, M, M	p.Ala435Ser, p.Ala375Ser, p.Ala375Ser, p.Ala375Ser, p.Ala375Ser, p.Ala380Ser	165	0.05
17	57220268	C	CA	AASDH	ENST00000205214, ENST00000513376, ENST00000451613, ENST00000602986, ENST00000502617	H, H, H, H, H	p.Leu440fs, p.Leu340fs, p.Leu440fs, p.Leu287fs, p.Leu440fs	76	0.09

17	62598522	A	T	LPHN3	ENST00000506720, ENST00000512091, ENST00000514591, ENST00000506700, ENST00000509896, ENST00000511324, ENST00000545650, ENST00000507164, ENST00000508693, ENST00000507625, ENST00000504896, ENST00000514157, ENST00000506746, ENST00000508946, ENST00000514996	M, M, M, M, M, M, M, M, M, M, M, M, M, M, M	p.Phe217Ile, p.Phe149Ile, p.Phe149Ile, p.Phe149Ile, p.Phe217Ile, p.Phe217Ile, p.Phe149Ile, p.Phe217Ile, p.Phe217Ile, p.Phe217Ile, p.Phe149Ile, p.Phe149Ile, p.Phe217Ile, p.Phe149Ile, p.Phe149Ile	165	0.33
17	83348356	A	C	HNRNPDL	ENST00000295470, ENST00000349655, ENST00000602300, ENST00000502762	M, M, M, M	p.Arg336Ser, p.Arg217Ser, p.Arg217Ser, p.Arg336Ser	76	0.05
17	85623541	T	G	WDFY3	ENST00000295888, ENST00000322366, ENST00000514711	M, M, M	p.Pro2854Gln, p.Pro2837Gln, p.Pro457Gln	76	0.05
17	8603122	A	G	CPZ	ENST00000360986, ENST00000315782	M, M	p.Ala132Thr, p.Ala121Thr	165	0.41
17	90833127	A	T	MMRN1	ENST00000508372	H	p.Met1?	1253	0.06
18	138857004	A	C	TMEM173	ENST00000330794	H	p.Glu286*	117	0.06
18	140735310	C	CG	PCDHGA4	ENST00000571252	H	p.Val182fs	337	0.06
18	14287144	T	A	TRIO	ENST00000344204, ENST00000537187, ENST00000512070, ENST00000509967	M, M, M, M	p.Asn171Ile, p.Asn171Ile, p.Asn112Ile, p.Asn122Ile	260	0.06
18	38425105	T	G	EGFLAM	ENST00000354891, ENST00000322350, ENST00000336740, ENST00000354891, ENST00000322350, ENST00000336740	M, M, M, M, M, M	p.Cys574Phe, p.Cys574Phe, p.Cys340Phe, NA, NA, NA	1260	0.07

18	56526955	A	G	GPBP1	ENST00000424459, ENST00000506184, ENST00000454432, ENST00000511209, ENST00000264779, ENST00000538707	H, H, H, H, H, H	p.Trp73*, p.Trp73*, p.Trp73*, p.Trp80*, p.Trp80*, p.Trp80*	76	0.06
18	57753207	G	C	PLK2	ENST00000274289, ENST00000502671, ENST00000503115, ENST00000515415, ENST00000509555	H, H, H, H, H	NA, NA, NA, NA, NA	165	0.16
18	67591068	CTGAGTATCGAGAAATTGACAAACGTATGAACAG	C	PIK3R1	ENST00000521381, ENST00000521657, , ENST00000396611, ENST00000521381, ENST00000521657, ENST00000274335, ENST00000320694, ENST00000336483, ENST00000523872	M, H, H, M, M, M, M	p.Ser565_Ile566insGluTyrArgGluIleAspLysArgMetAsnSer, p.Ser565_Ile566insGluTyrArgGluIleAspLysArgMetAsnSer, p.Ser565_Ile566insGluTyrArgGluIleAspLysArgMetAsnSer, p.Ser565_Ile566insGluTyrArgGluIleAspLysArgMetAsnSer, p.Ser265_Ile266insGluTyrArgGluIleAspLysArgMetAsnSer, p.Ser295_Ile296insGluTyrArgGluIleAspLysArgMetAsnSer, p.Ser202_Ile203insGluTyrArgGluIleAspLysArgMetAsnSer	115	0.38
18	71491013	T	G	MAP1B	ENST00000296755, ENST00000511641, ENST00000504492	H, H, H	p.Glu611*, p.Glu628*, p.Glu485*	1253	0.06
18	74028851	T	C	GFM2	ENST00000296805, ENST00000345239, ENST00000509430	M, M, M	p.Gly528Glu, p.Gly481Glu, p.Gly528Glu	117	0.06

18	98235356	G	C	CHD1	ENST00000284049	H	p.Ala305Pro	448	0.06
19	123319065	A	G	CLVS2	ENST00000275162	M	p.Arg48His	165	0.08
19	129371125	A	G	LAMA2	ENST00000421865	M	p.Gly59Arg	117	0.05
19	146351171	G	A	GRM1	ENST00000282753, ENST00000361719, ENST00000392299, ENST00000492807, ENST00000355289, ENST00000507907	M, M, M, M, M, M	p.Asn173Ser, p.Asn173Ser, p.Asn173Ser, p.Asn173Ser, p.Asn173Ser, p.Asn173Ser	100	0.06
19	30545688	T	C	ABCF1	ENST00000326195, ENST00000376545, ENST00000396515, ENST00000441867	H, H, H, H	p.Gln72*, p.Gln72*, p.Gln72*, p.Gln72*	76	0.07
19	75828810	T	C	COL12A1	ENST00000322507, ENST00000425443, ENST00000345356, ENST00000416123, ENST00000483888	M, M, M, M, M	p.Gly2435Ser, p.Gly73Ser, p.Gly1271Ser, p.Gly2435Ser, p.Gly2435Ser	1272	0.49
19	80223189	T	C	LCA5	ENST00000369846, ENST00000392959, ENST00000467898	M, M, M	p.Glu154Lys, p.Glu154Lys, p.Glu154Lys	337	0.08
19	84284785	A	G	SNAP91	ENST00000369694, ENST00000521485, ENST00000195649, ENST00000439399, ENST00000428679, ENST00000437520, ENST00000521743, ENST00000520302, ENST00000520213, ENST00000523448	H, H, H, H, H, H, H, H, H, H	p.Gln796*, p.Gln791*, p.Gln791*, p.Gln796*, p.Gln796*, p.Gln489*, p.Gln796*, p.Gln766*, p.Gln489*, p.Gln137*	260	0.06
19	90452967	T	C	MDN1	ENST00000369393, ENST00000428876	H, H	p.Trp1450*, p.Trp1450*	187	0.06

20	117232010	G	A	CFTR	ENST00000003084, ENST00000454343, ENST00000426809	M, M, M	p.Asn597Asp, p.Asn536Asp, p.Asn567Asp	1260	0.09
20	117304893	A	C	CFTR	ENST00000003084, ENST00000454343, ENST00000426809, ENST00000600166	M, M, M, M	p.Pro1372His, p.Pro1311His, p.Pro1342His, p.Pro80His	1253	0.2
20	121731881	A	G	AASS	ENST00000393376, ENST00000417368, ENST00000358954	M, M, M	p.Ser631Phe, p.Ser631Phe, p.Ser631Phe	115	0.34
20	138864387	A	G	TTC26	ENST00000464848, ENST00000430935, ENST00000495038, ENST00000478836, ENST00000343187	M, M, M, M, M	p.Cys437Tyr, p.Cys437Tyr, p.Cys306Tyr, p.Cys330Tyr, p.Cys406Tyr	117	0.05
20	140508693	A	C	BRAF	ENST00000288602, ENST00000497784	H, M	p.Gly203*, p.Gly188Trp	1253	0.05
20	142120102	A	C	TRBV7-7	ENST00000390377	M	p.Gly22Val	100	0.06
20	142364661	T	C	TRBV24-1	ENST00000390397	M	p.Ser99Phe	76	0.06
20	144094334	C	T	NOBOX	ENST00000467773, ENST00000223140, ENST00000483238	H, H, H	p.Ter692Trpext*?, p.Ter575Trpext*?, p.Ter660Trpext*?	165	0.06
20	150709519	A	G	NOS3	ENST00000297494, ENST00000461406	M, M	p.Arg1022Gln, p.Arg816Gln	337	0.07
20	151805315	A	G	GALNT11	ENST00000320311, ENST00000430044, ENST00000452146, ENST00000434507	H, H, H, H	p.Trp302*, p.Trp302*, p.Trp221*, p.Trp302*	76	0.05

20	154755458	T	C	PAXIP1	ENST00000397192, ENST00000404141	M, M	p.Gly638Ser, p.Gly638Ser	452	0.25
20	30961763	G	C	AQP1	ENST00000434909, ENST00000509504, ENST00000311813, ENST00000441328, ENST00000409899, ENST00000409611	M, M, M, M, M, M	p.Thr216Ser, p.Thr333Ser, p.Thr156Ser, p.Thr73Ser, p.Thr41Ser, p.Thr105Ser	1260	0.19
20	38284804	C	T	TRGC2	ENST00000436911	M	p.Asn119Ser	448	0.17
20	81350145	T	C	HGF	ENST00000222390, ENST00000457544	M, M	p.Gly396Asp, p.Gly391Asp	452	0.05
20	99490319	A	G	TRIM4	ENST00000355947, ENST00000349062	H, H	p.Gln324*, p.Gln298*	165	0.06
21	100115217	C	A	VPS13B	ENST00000358544, ENST00000355155, ENST00000496144, ENST00000395996, ENST00000357162, ENST00000441350	M, M, M, M, M, M	p.Asn150Thr, p.Asn150Thr, p.Asn150Thr, p.Asn150Thr, p.Asn150Thr, p.Asn150Thr	1272	0.48
21	141745383	T	C	PTK2	ENST00000517887, ENST00000522684, ENST00000519654, ENST00000535192, ENST00000519465, ENST00000521059, ENST00000395218, ENST00000523539, ENST00000538769, ENST00000340930, ENST00000519419, ENST00000521986, ENST00000521981	M, H, M, M, M, H, H, M, M, H, M, M, M	p.Arg710Gln, p.Arg666Gln, p.Arg676Gln, p.Arg666Gln, p.Arg294Gln, p.Arg666Gln, p.Arg666Gln, p.Arg338Gln, p.Arg334Gln, p.Arg666Gln, p.Arg710Gln, p.Arg364Gln, p.Gly104Ser	1253	0.33
21	143558851	A	G	BAI1	ENST00000323289, ENST00000517894, ENST00000521208	M, M, M	p.Gly443Asp, p.Gly443Asp, p.Gly443Asp	337	0.07
21	16961933	G	A	MICU3	ENST00000318063, ENST00000519044	M, M	p.Thr340Ala, p.Thr184Ala	117	0.06

21	17412484	A	G	SLC7A2	ENST00000470360, ENST00000398090	M, M	p.Arg402Gln, p.Arg402Gln	115	0.05
21	25317947	T	A	CDCA2	ENST00000330560, ENST00000380665	H, H	p.Lys37*, p.Lys22*	448	0.08
21	31497697	A	C	NRG1	ENST00000520407	M	p.Pro66Gln	452	0.05
21	87242277	T	C	SLC7A13	ENST00000297524, ENST00000419776	M, M	p.Ser77Asn, p.Ser77Asn	1260	0.07
21	94827630	A	C	TMEM67	ENST00000453321, ENST00000409623, ENST00000323130	H, H, H	p.Cys954*, p.Cys873*, p.Cys944*	100	0.06
22	108397506	A	G	FKTN	ENST00000223528, ENST00000602661	H, H	p.Trp449*, p.Trp449*	117	0.08
22	124921954	T	C	NDUFA8	ENST00000373768, ENST00000537618	H, H	p.Met1?, p.Met1?	448	0.05
22	138670610	T	G	KCNT1	ENST00000298480, ENST00000487664, ENST00000371757, ENST00000486577, ENST00000491806, ENST00000488444, ENST00000490355, ENST00000263604	M, M, M, M, M, M, M, M	p.Ala891Ser, p.Ala846Ser, p.Ala891Ser, p.Ala850Ser, p.Ala858Ser, p.Ala872Ser, p.Ala870Ser, p.Ala872Ser	1272	0.05
22	37887862	C	A	SLC25A51	ENST00000242275, ENST00000380590, ENST00000377716	M, M, M	p.Phe229Cys, p.Phe229Cys, p.Phe229Cys	1260	0.06
22	70918627	C	G	FOXD4L3	ENST00000342833	M	p.Ala254Pro	115	0.09
22	70918670	C	T	FOXD4L3	ENST00000342833	M	p.Leu268Pro	165	0.12

22	98691049	T	C	ERCC6L2	ENST00000288985, ENST00000437817, ENST00000456993, ENST00000426805	H, H, H, H	p.Arg563*, p.Arg374*, p.Arg245*, p.Arg50*	452	0.05
X	110494902	A	C	CAPN6	ENST00000541758	H	p.Met1?	76	0.07
X	110929363	G	A	ALG13	ENST00000473389, ENST00000492038	H, H	NA, NA	76	0.08
X	151336771	T	A	GABRA3	ENST00000370314, ENST00000535043	M, M	p.Phe470Ile, p.Phe470Ile	187	0.06
X	153363099	C	CGCG	MECP2	ENST00000453960, ENST00000369957	M, M	p.Ala8del, p.Ala7del	337	0.14
X	153995301	T	C	DKC1	ENST00000369550, ENST00000413910, ENST00000437719, ENST00000412124	M, M, M, M	p.His160Tyr, p.His160Tyr, p.His145Tyr, p.His4Tyr	117	0.07
X	154130395	A	G	F8	ENST00000360256	M	p.Arg2016Trp	117	0.05
X	2833605	T	C	ARSD	ENST00000381154	H	p.Trp331*	337	0.33
X	41200735	G	A	DDX3X	ENST00000399959, ENST00000478993, ENST00000457138, ENST00000441189, ENST00000542215	H, H, H, H, H	NA, NA, NA, NA, NA	165	0.89
X	47426704	A	G	ARAF	ENST00000377045	M	p.Gly317Arg	117	0.1
X	49957846	T	G	AKAP4	ENST00000358526	M	NA	1260	0.06
X	51487728	A	G	GSPT2	ENST00000340438	M	p.Ala336Thr	448	0.06

X	53440089	T	C	SMC1A	ENST00000322213, ENST00000463684, ENST00000428014	H, H, H	NA, NA, NA	165	0.08
X	66937392	T	C	AR	ENST00000374690, ENST00000396043	M, M	p.Ala749Val, p.Ala217Val	100	0.08
X	85211351	A	T	CHM	ENST00000357749, ENST00000537751	H, H	p.Lys325*, p.Lys177*	448	0.14
Y	23545593	A	G	CYorf17	ENST00000382764	M	p.Pro60Leu	76	0.14
